# Adaptive laboratory evolution of *Clostridium autoethanogenum* to metabolize CO_2_ and H_2_ enhances growth rates in chemostat and unravels proteome and metabolome alterations

**DOI:** 10.1111/1751-7915.14452

**Published:** 2024-04-03

**Authors:** James Heffernan, R. Axayactl Garcia Gonzalez, Vishnu Mahamkali, Tim McCubbin, Dara Daygon, Lian Liu, Robin Palfreyman, Audrey Harris, Michael Koepke, Kaspar Valgepea, Lars Keld Nielsen, Esteban Marcellin

**Affiliations:** ^1^ Australian Institute of Bioengineering and Nanotechnology The University of Queensland St. Lucia Queensland Australia; ^2^ ARC Centre of Excellence in Synthetic Biology The University of Queensland St. Lucia Queensland Australia; ^3^ LanzaTech Inc. Skokie Illinois USA; ^4^ Queensland Metabolomics and Proteomics Q‐MAP The University of Queensland St. Lucia Queensland Australia; ^5^ ERA Chair in Gas Fermentation Technologies, Institute of Technology University of Tartu Tartu Estonia; ^6^ The Novo Nordisk Foundation Center for Biosustainability Technical University of Denmark Kgs. Lyngby Denmark

## Abstract

Gas fermentation of CO_2_ and H_2_ is an attractive means to sustainably produce fuels and chemicals. *Clostridium autoethanogenum* is a model organism for industrial CO to ethanol and presents an opportunity for CO_2_‐to‐ethanol processes. As we have previously characterized its CO_2_/H_2_ chemostat growth, here we use adaptive laboratory evolution (ALE) with the aim of improving growth with CO_2_/H_2_. Seven ALE lineages were generated, all with improved specific growth rates. ALE conducted in the presence of 2% CO along with CO_2_/H_2_ generated Evolved lineage D, which showed the highest ethanol titres amongst all the ALE lineages during the fermentation of CO_2_/H_2_. Chemostat comparison against the parental strain shows no change in acetate or ethanol production, while Evolved D could achieve a higher maximum dilution rate. Multi‐omics analyses at steady state revealed that Evolved D has widespread proteome and intracellular metabolome changes. However, the uptake and production rates and titres remain unaltered until investigating their maximum dilution rate. Yet, we provide numerous insights into CO_2_/H_2_ metabolism via these multi‐omics data and link these results to mutations, suggesting novel targets for metabolic engineering in this bacterium.

## Graphical abstract

Adaptive laboratory evolution was used in this study to improve *Clostridium autoethanogenum*'s growth on CO_2_ and H_2_. The growth rate improved using various methods, where one using a 2% CO gas mixture retained the most ethanol produciton – Evolved D. Under the same CO_2_ and H_2_ chemostat conditions, no changes in rates and titres were found between the wildtype and Evolved D, but widespread proteome and metabolome changes appear to allow higher growth rates when required.

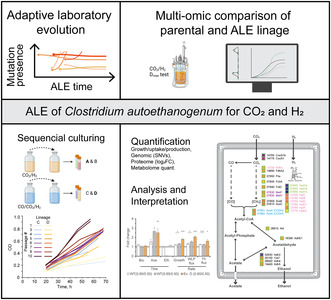

## INTRODUCTION

To achieve the goals set in the Sustainable Development Goals (SDGs 7 and 13) (United Nations, 2021), reductions in our greenhouse gas emissions need to be larger than 2020's drop in emissions resulting from the COVID‐19 pandemic (Liu, Ciais, et al., 2020). Various avenues for valorizing C1 compounds are being investigated, where biotechnologies offer unique beneficial properties, such as scalability and flexibility with renewable integration (Clomburg et al., 2017; Liu, Wang, et al., 2020; Molitor et al., 2019). Chemolithotroph metabolism of (point‐source) carbon waste achieves overall negative carbon emissions (Haas et al., 2018; Handler et al., 2016; Liew et al., 2022; Wood et al., 2021) without the need for large amounts of other resources (e.g., sugar) or direct light. Gas‐to‐liquid fermentation demonstrates appropriate efficiency, sustainability and feasibility of commercialization for such a process (Haas et al., 2018; Handler et al., 2016), and LanzaTech has shown its economic feasibility with several industrial‐scale CO‐to‐ethanol plants utilizing acetogenic bacteria (Fackler et al., 2021).

Acetogens, referring to the microbe's native ability to produce acetyl‐CoA from CO_2_, are anaerobic Clostridia that demonstrate flexibility in metabolizing various C1 oxide compounds (Heffernan et al., 2020; Kim et al., 2021; Novak et al., 2021; Valgepea et al., 2018). The reductive acetyl‐CoA/Wood–Ljungdahl pathway (WLP) is their key autotrophic pathway, the most efficient CO_2_ fixation pathway currently known (Liu, Wang, et al., 2020). Furthermore, as numerous acetogens are amenable to metabolic engineering with genetic engineering toolboxes (Fackler et al., 2021), an ideal characteristic of platform C1 biocatalysts (Bae et al., 2022; Karim et al., 2020; Liew et al., 2022; Pavan et al., 2022), it is possible to establish production of various chemicals (Simpson & Köpke, 2020), develop understanding of their metabolism and, in combination with systems biology tools (Heffernan et al., 2022), optimize them for industrial‐scale biomanufacturing (Liew et al., 2022). So far, the typical C1 fermentation is based on gases with a high CO component (i.e., syngas), as CO is capable of being both the carbon and energy source. CO_2_ can be the sole carbon source of acetogen fermentation, but it is more restrictive than CO‐based fermentation due to the lack of energy contained in CO_2_ (Bertsch & Müller, 2015; Emerson & Stephanopoulos, 2019; Wood et al., 2021), with reductive energy being supplied by hydrogen or electrons (Claassens et al., 2019; Simpson & Köpke, 2020). Regardless, effective recycling of CO_2_ with renewable energy ideally mitigates its polluting effect on the climate (Almeida Benalcázar et al., 2022; García & Galán, 2022).

Improving C1 fermentation typically involves enhancing physicochemical properties (e.g., gas–liquid mass transfer) and optimizing metabolism (Bae et al., 2022), where comparing CO_2_ fermenting organisms also generates understanding of metabolism (Bae et al., 2022; Bengelsdorf et al., 2018; Groher & Weuster‐Botz, 2016). Furthermore, CO_2_/H_2_, CO/H_2_ or CO metabolisms have been improved or optimized through co‐utilization of supplementary substrates, notably CO_2_/H_2_ supplemented with (+) CO (Heffernan et al., 2020; Novak et al., 2021), CO_2_/H_2_ + formate (Neuendorf et al., 2021), CO_2_/H_2_ + nitrate (Emerson et al., 2019), CO + arginine (Valgepea, Loi, et al., 2017), CO_2_/H_2_ + glucose (Park et al., 2019) and CO/H_2_ + fructose (Jones et al., 2016). However, few of these are tested in chemostats (Heffernan et al., 2020), which are the only industrially relevant and reproducible fermentation mode to test uptake and production rates (Heffernan et al., 2022). Based on metabolism and technology developments (e.g., genetic/metabolic engineering and system biology), *Clostridium autoethanogenum* and closely related *Clostridium ljungdahlii* have advantageous traits for industrial biomanufacturing from C1 gases (Fackler et al., 2021). We recently characterized *C. autoethanogenum*'s CO_2_/H_2_ growth under chemostat conditions, demonstrating good ethanol production (Heffernan et al., 2020). However, there are limited investigations on strategies for improving CO_2_ fermentation via genetic modification.

Genetic studies of *C. autoethanogenum* DSM 10061 fermenting CO_2_/H_2_ have shown that knockout of specific carbon monoxide dehydrogenase isoenzymes can improve batch growth (Liew et al., 2016), and knockout of acetaldehyde: ferredoxin oxidoreductases (AOR) reduced batch growth and ethanol production (Liew et al., 2017). However, an understanding of *C. autoethanogenum*'s metabolism is still developing, and many highly expressed proteins remain functionally unclear (Valgepea et al., 2022). Adaptive laboratory evolution (ALE) is a convenient method for interrogating metabolism and improving fermentation characteristics. For native C1 metabolism, implementing ALE improved methanol assimilation in *Saccharomyces cerevisiae* (Espinosa et al., 2020), tolerance to and growth with CO in *Eubacterium limosum* (Kang et al., 2020), growth with syngas in *Rhodospirillum rubrum* (Hernández et al., 2016), growth and acetate production from CO_2_ in *Sporomusa ovata* (via methanol ALE) (Tremblay et al., 2015), and growth and product formation from CO_2_ in *Clostridium carboxidivorans* (Antonicelli et al., 2023). In this work, we used ALE to obtain seven mutant lineages with improved growth on CO_2_/H_2_. We performed a variant analysis to identify critical mutations leading to the improved phenotype. Proteomics and metabolomics identified novel pathways that contribute to the enhanced phenotype. The combination of genomics, proteomics and metabolomics shows how multi‐omics approaches can be used to understand phenotype–genotype relationships in carbon recycling biotechnology processes, contributing to avenues for combating climate change.

## EXPERIMENTAL PROCEDURES

### Bacterial strains

A non‐commercial derivate of *C. autoethanogenum* DSM 10061 strain – DSM 19630 – deposited in the German Collection of Microorganisms and Cell Cultures (DSMZ) was used as starting culture for all experiments. All strains were stored as glycerol stocks at −80°C. Cells were grown under strictly anaerobic conditions at 37°C.

### Bottle evolution

Adaptive laboratory evolution (ALE) was performed in duplicate serum bottles (125 mL) where cultures grew in 25 mL PETC‐MES medium without bicarbonate or fructose (Tremblay et al., 2015) and pressurized to ~190 kPa with CO_2_/H_2_ or CO/CO_2_/H_2_ (~23% CO_2_, 67% H_2_ and 10% Ar or ~2% CO, 23% CO_2_, 65% H_2_ and 10% Ar; BOC Australia). ALE parameters – passage size (1%) and optical density (>50% of expected maximum) and termination criteria – were based mainly on LaCroix et al. (2015, 2017).

### Chemostat testing

Autotrophic chemostat cultures grew at 37°C in chemically defined medium (Valgepea, de Souza Pinto, et al., 2017), maintained at pH 5 by 5 M NH_4_OH, fed with 32 mL/min CO_2_/H_2_ (~23% CO_2_, ~67% H_2_ and ~10% Ar; BOC Australia) and stirred at 500 rpm. Gas flow rates and stirring speed were increased up to 50 mL/min and 1000 rpm, respectively, when testing maximum dilution rate. Steady states were achieved at dilution rates (D) = 0.50 (DSM 19630 and Evolved D), 0.60, 0.65 (DSM 19630) and 0.90 ± 0.01 day^−1^ (Evolved D) (specific growth rates [μ] = 0.0203 ± 0.0008, 0.0211 ± 0.0003, 0.0247 ± 0.0004, 0.0269 ± 0.0002 and 0.0384 ± 0.0001 h^−1^, respectively [average ± standard deviation]). Bioreactor off‐gas was analysed by an online Hiden HPR‐20‐QIC mass spectrometer. The Faraday cup detector monitored the intensities of H_2_, ethanol, H_2_S, Ar and CO_2_ at 2, 31, 34, 40 and 44 atomic mass units (amu), respectively, in the bioreactor off‐gas. See Heffernan et al. (2020) for further details.

### Biomass and extracellular metabolite analysis

Biomass concentration (gDCW/L) was estimated by measuring the OD of the culture at 600 nm and using a correlation coefficient of 0.21 between culture OD and dry cell weight determined in Valgepea et al. (2018). Extracellular metabolome analysis was carried out using HPLC methods described in Valgepea, de Souza Pinto, et al. (2017).

### Whole‐population genome sequencing and analysis

The parental, Evolved A, D, E and G strains were analysed for comparative genomics. 200 mL cultures were grown in PETC‐MES medium without bicarbonate, fructose or yeast extract with CO_2_/H_2_ (~140 kPa) and pelleted at the end of the exponential phase (4°C, 20,000 *g*). Cell pellets were washed with cold 10% sucrose 3× and then stored at −80°C. DNA extraction, library preparation and sequencing were performed by the Australian Centre for Ecogenomics (ACE). Briefly, typical methods were used for short‐read sequencing, returning 3.4, 3.4, 2.7, 3.1 and 2.5 Gbp of reads for parental, Evolved A, D, E and G sequencing – achieving ~35 mean mapping quality for forward and reverse reads (Figure S1A). Sequencing QC, alignments and validation (Figure S1B) were performed via the Australian instance of Galaxy (Afgan et al., 2018). Variants were identified using Snippy (Seemann, n.d.) under default settings, where alignments achieved ~500× coverage (Figure S1C). Large mutations to the genome were also identified through analysis of the per‐base coverage from QualiMap (Okonechnikov et al., 2016). See full details of sequencing in Appendix S1.

### Intracellular metabolomic analysis

Chemostat cultures were pelleted by immediate centrifugation (20,000 *g* for 2 min at 4°C) followed by resuspension in ice‐cold 50% acetonitrile to extract intracellular metabolites. Cellular debris was removed by centrifugation, and the supernatant was stored at −80°C. Next, samples were freeze‐dried and concentrated in 2% acetonitrile with 5 μM AZT as internal standard. Sample clean‐up was performed by centrifugal filtration (14,000 *g* for 15 min at 4°C) through Amicon® Ultra (0.5 mL, 3 kDa) columns.

Metabolites were analysed using liquid chromatography–tandem mass spectrometry (LC‐MS/MS) performed using a Shimadzu Nexera X2 UHPLC system coupled to a Shimadzu 8060 triple quadrupole mass spectrometer. Chromatographic separation was achieved on a Phenomenex Gemini NX‐C18 column (00A‐4453‐B0) with guard column (SecurityGuard Gemini NX‐C18, 4 × 2 mm), operated at 45°C at a flow rate of 300 μL/min. Mobile phase A was 7.5 mM tributylamine aqueous solution (pH to 4.95 with acetic acid), and mobile phase B was acetonitrile. Mass spectrometry was achieved using the scheduled multiple reaction monitoring (sMRM) method on the negative ionization mode using transient ions as previously published (Espinosa et al., 2020). Concentrations of each metabolite were calculated based on standard curves from serial dilutions of authentic chemical standards (Sigma). Collected data were processed using LabSolutions InsightTM (Shimadzu).

### Proteomic analysis

All samples were taken at the end of their respective steady states. In brief, peptides were extracted using a typical S‐Trap (ProtiFi) protocol and then analysed by liquid chromatography–mass spectrometry (LC‐MS). ‘Evolved D vs. DSM 19630’ and ‘CO/CO_2_/H_2_ vs. CO_2_/H_2_’ comparative proteomic analyses were performed using different variations of methods for extraction, mass spectrometry and data analysis (data‐independent acquisition [DIA] and data‐dependent acquisition [DDA], respectively). Proteins were identified as differentially expressed (DE) using cut‐off thresholds of 1% false discovery rate, *p*‐value ≤0.05, *q*‐value ≤0.05, number of unique peptides ≥2 and fold change (FC) ≥ 1.5 or ≤0.667 (i.e., |log_2_FC| ≥ 0.585). Full details of proteomic analysis are shown in Appendix S1. The DDA and DIA proteomic data have been deposited to the ProteomeXchange Consortium via the PRIDE (Perez‐riverol et al., 2022) partner repository with the dataset identifiers PXD048075 and PXD048143, respectively. The result output of ‘Evolved D vs. DSM 19630’ (DIA) can also be found in Table S1.

## RESULTS

### *C. autoethanogenum* evolved with supplemental CO grows faster on CO_2_/H_2_ while maintaining ethanol production

**FIGURE 1:**
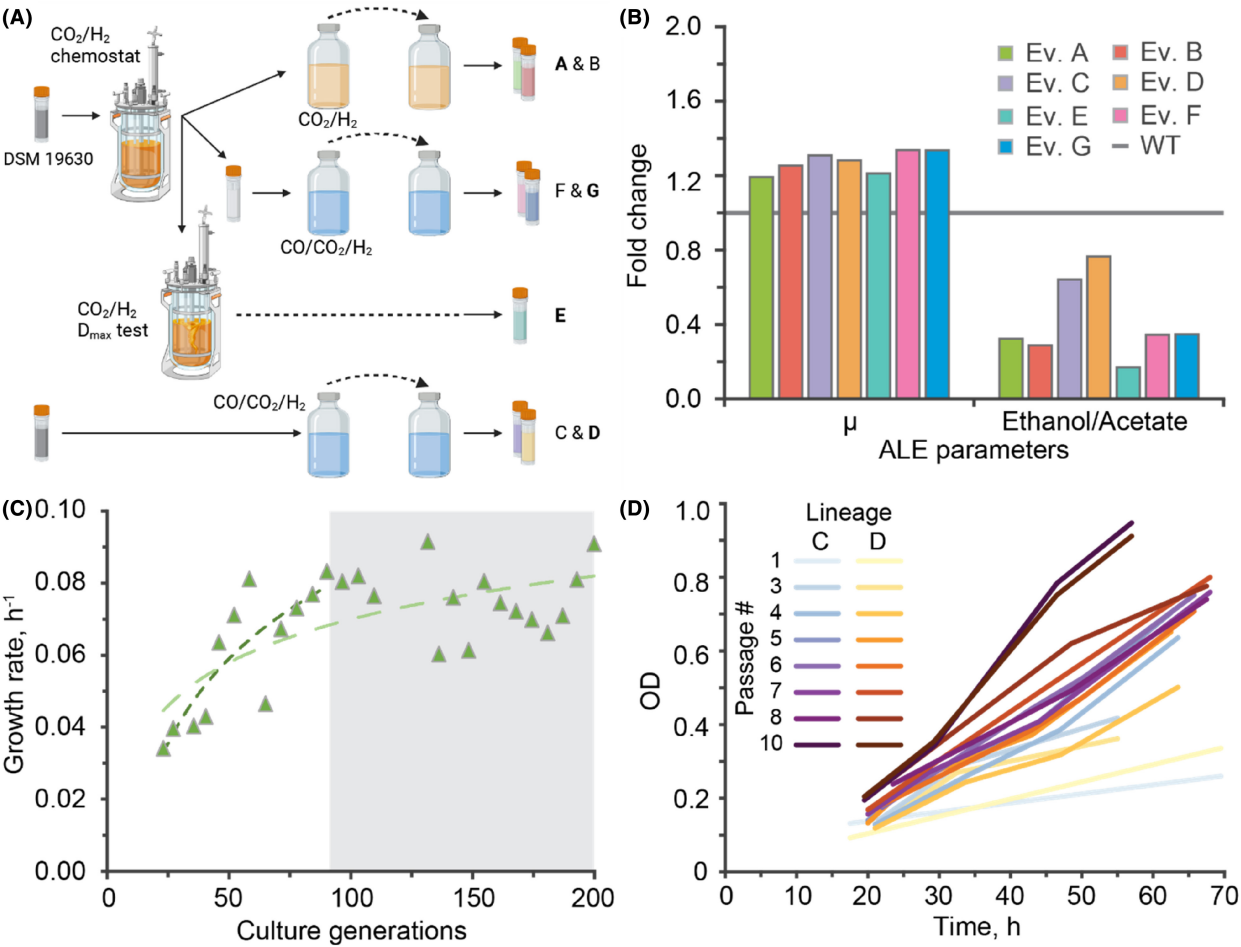
Summary of *Clostridium autoethanogenum* ALE and quantification prior to chemostat experiments. (A) An illustration of the ALE method for each lineage. Created with BioRender.com. (B) Initial comparison of Evolved (Ev.) lineages when grown on CO_2_/H_2_ to investigate ALE parameters. Fold change of μ and ethanol/acetate ratio represents individual cultures (*n* = 1) compared to the parental strain (WT, indicated by horizontal line). See Figure S2D–G for associated batch profiles and further ALE information. (C) Example of increase in specific growth rate with increasing culture generation; each point represents a single bottle of Evolved A growth. The grey box (~90–200 h) indicates a period of minimal change in specific growth rate, also shown by fitted logarithmic curves. (D) Example of ALE growth curves from Evolved C and D lineages. Ten passages shown for clarity.

**FIGURE 2:**
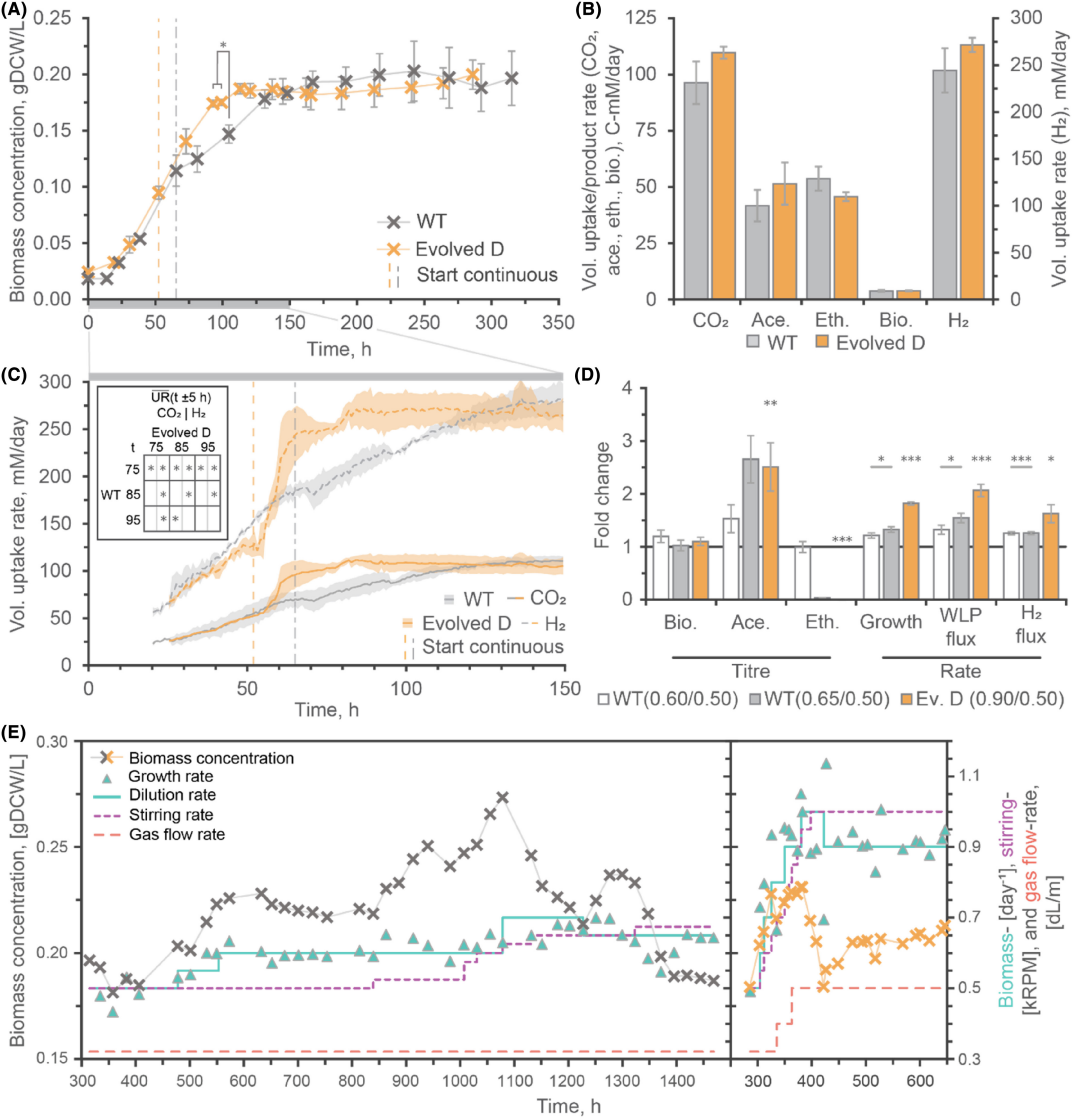
Bioreactor data comparing *Clostridium autoethanogenum* DSM 19630 (WT) and Evolved D fermenting CO_2_/H_2_. (A) Growth profiles of WT and Evolved D up to the end of their first chemostat steady state. (B) Volumetric uptake and production rates of WT and Evolved D during a steady‐state chemostat operated at 0.50 day^−1^ dilution rate. (C) Gas uptake rates (CO_2_ and H_2_) leading up to the first steady state; grey boxes show time scale compared to (A). Insert shows significant differences for average uptake rates over a 10‐h period (measurements every ~1 h). (D) Changes to titres (C‐mM) and rates ([C‐]mmol gDCW^−1^ day^−1^) were caused by increasing dilution rate for WT and Evolved D steady states. WLP fluxes are based on the average of uptake (CO_2_) and production (biomass, acetate and ethanol) due to limited gas data for Evolved D steady state at 0.90 day^−1^ (MS crash; see Figure S3). (E) DSM 19630 and Evolved D growth during maximum dilution rate test shown as single bioreactors (*n* = 1) are displayed. Data at dilution rate 0.5 day^−1^ (A–C) and Evolved D at 0.90 day^−1^ (D) represent the average ± standard deviation (SD) of three and two biological replicates, respectively (*n* = 3 and 2), while WT at 0.60 and 0.65 day^−1^ represent individual replicates (*n* = 1) ± SD during steady state. Statistics for WT steady states >0.50 day^−1^ are performed using both steady states (combined for *n* = 2), indicated by horizontal bars under *p*‐value indicators. Differences meeting *p*‐value thresholds are represented as follows: <0.05*, <0.01** and <0.001***. See Figure S3 for further details of steady states >0.50 day^−1^. Ace., acetate; Bio., biomass; Eth., ethanol; Ev., Evolved; Vol., volumetric.

Two independent lineages underwent ALE with CO_2_/H_2_, inoculated with *C. autoethanogenum* DSM 19630 from CO_2_/H_2_ steady‐state chemostat cultures (Heffernan et al., 2020) (dilution rate [D] = 0.5 day^−1^, Figure 1A). After ~90 generations, the maximum specific growth rate (μ) of these lineages increased ~1.22‐fold (Figure 1B), while a further ~100 generations did not facilitate notable improvement in growth (Figure 1C; Figure S2A,B). Glycerol stocks of the lineages at ~90 generations were used in all subsequent experiments (named hereafter Evolved A and B). However, we noted that ethanol/acetate production decreased to ~0.31‐fold after ALE (Figure 1B), probably due to energetic pressures associated with ethanol production (Mock et al., 2015) – as acetate production leads to 0.25 ATP/H_2_ compared to 0.20 ATP/H_2_ for ethanol. For further comparison of lineages, glycerol stocks from CO_2_/H_2_ chemostat tests of DSM 19630 at maximum specific growth rate (dilution rate [D_max_], Figure 2E; Figure S3) were also used for comparison (named hereafter Evolved E). This lineage showed similar growth characteristics to Evolved A and B, but with lower ethanol/acetate titre (Figure 1B). These results suggested that a change in the ALE method was necessary to maintain ethanol production while improving the growth rate. We previously demonstrated that minor supplementation of CO (2%) enhanced *C. autoethanogenum* growth in chemostats (Heffernan et al., 2020) (Figure S4A,B shows the increase in bottle growth also). Subsequently, we hypothesized that ethanol production could be retained during ALE for improved growth rate if we used CO‐supplemented CO_2_/H_2_ (CO/CO_2_/H_2_). Two independent lineages underwent ALE with CO/CO_2_/H_2_ inoculated with DSM 19630 (named hereafter Evolved C and D, Figure 1D). Evolved C and D maintained ethanol production (~0.70‐fold ethanol/acetate, Figure 1B) and had a ~1.30‐fold increase in specific growth rate after ~95 generations (Figure S2C). In parallel, glycerol stocks from CO_2_/H_2_ steady‐state chemostat (Heffernan et al., 2020) were also grown on CO/CO_2_/H_2_ (named hereafter Evolved F and G). These lineages had ~1.34‐fold greater μ, ~1.92‐fold and ~0.6‐fold acetate and ethanol after ~95 generations of ALE (Figure 1; Figure S2).

Acetate concentration (Figure S2E,G) and μ changes are similar for Evolved A and B and Evolved C and D (for *n* = 2). However, CO/CO_2_/H_2_ ALE (Evolved C and D) had ~2‐fold ethanol concentration at the end of exponential growth compared to CO_2_/H_2_ ALE (Evolved A and B, *p*‐value = 0.049). These initial comparisons were performed as single, large‐bottle fermentations because quantitative comparison would be performed in replicate bioreactors and genomic analysis was used to explore these initial results further. One lineage from each ALE method (Evolved A, D and G) was selected for sequencing. Evolved E was also sequenced due to enhanced growth characteristics compared to DSM 19630. Minimal mutations in the genome were observed for the selected lineages (Table 1). However, many mutations were conserved across the lineages. Mutations were involved in transcription and translation (*perR*, *argR* and *prfB*), amino acid synthesis ([*argR*] *thrB*) and cell growth (*gerKA*) – cellular functions are KEGG Orthology (KO) Level 2 from Valgepea et al. (2022). Furthermore, genes within 500 base pairs of the mutations were involved in base excision repair (*recJ2*), transport (*TC.APA7*, *gutA* and MFS transporter[s]), metabolite synthesis (CAETHG_RS030805 and CAETHG_RS13815) and a solute‐binding protein (CAETHG_RS07045). More thorough information about these genes and their variants is given in Appendices S2 and S3.

**TABLE 1:** Summary of mutations by lineage.

Gene	Position	Mutation (DNA) [AA]	Ev.A	Ev.D	Ev.E	Ev.G
ncDNA	710,848	C → A	+			
*perR*	1,565,578	(205/423: G → A) [69/140: Glu → Lys]			+	+
	1,565,629	(256/423: C → A) [86/140: His → Asn]	+		+	+
*gerKA*	1,606,333	(1537/1578: C → A) [513/525: Gln → Lys]		+		
*prfB*	2,525,941	(463/1107: A → T) [155/368: Arg → *]			+	+
	2,526,452	(974/1107: G → T) [325/368: Arg → Ile]		+		
*thrB*	3,047,148	(791/900: A → G), [264/299: Asn → Ser]		+		
*argR*	3,278,628	(271/456: AATTT → TATTC), [91/151: AsnPhe → TyrSer]	+			+
	~3,278,550	DEL (~Δ bp)	70	15	70	70

*Note*: See Tables S2 and S3 for further details.

### ALE improved the maximum specific growth rate in bioreactors

The best performing lineage (Evolved D) was selected for a multi‐omics comparative analysis in bioreactors. Initially, few changes were observed in the bioreactors using conditions from the chemostat operation of DSM 19630 previously reported (Heffernan et al., 2020). No significant differences occurred during the batch phase, where both strains had a specific growth rate of ~0.022 h^−1^. However, at the onset of continuous operation (D = ~0.5 day^−1^ [0.021 h^−1^]) the cultures growth phases' shift and the specific growth rate of Evolved D was up to 1.43‐fold ±0.29 (n.s., *p* = 0.084) of WT at similar points after continuous ($\Delta t_{\text{continuous}}$ = ~20 and 16 h for Evolved D and WT, respectively). Furthermore, the biomass concentration of Evolved D was significantly higher during this phase (1.27‐fold ±0.08, $\Delta t_{\text{continuous}}$ = ~40 h, *p* = 0.038, Figure 2A). Gas uptakes also show differences between the start of continuous operation and steady state (Figure 2C). Yet, under the same (steady) chemostat conditions, no significant changes in rates or titres are observable (Figure 2A,B, Table S4). Therefore, intracellular metabolomic and proteomic samples were taken for further analysis.

To test for differences in robustness, we searched for conditions where the strains had varied production or growth rates (Mahamkali et al., 2020). At a dilution rate of 0.70 day^−1^, DSM 19630 could not maintain a steady state (washout – see Figure 2E; Figure S3). The maximum steady state was at 0.65 day^−1^ (with another at 0.60 day^−1^, Table S5). However, Evolved D grew steadily at 0.90 day^−1^, but washed out at a dilution rate of 1.00 day^−1^ (Figure 2E; Figure S3 and Table S5). Consistent with the observable differences at the onset of continuous operation, this suggests a 1.38‐fold increase in μ for Evolved D under chemostat conditions (Figure 2D). Increasing the dilution rate increased the WLP (CO_2_) and H_2_ flux for both Evolved D and its parental strain (Figure 2D), where the maximum steady‐state WLP flux of Evolved D was ~1.5‐fold its parental strain (*p*‐value = 0.036) and H_2_ was ~1.4‐fold (n.s., *p* = 0.063; see Figure 2D and Table S5). Proteomic and intracellular metabolomic samples were analysed to understand the new phenotype of Evolved D at the subcellular level.

### Proteome adaptations of ALE suggest enhanced C1 fixation and energy metabolism

**FIGURE 3:**
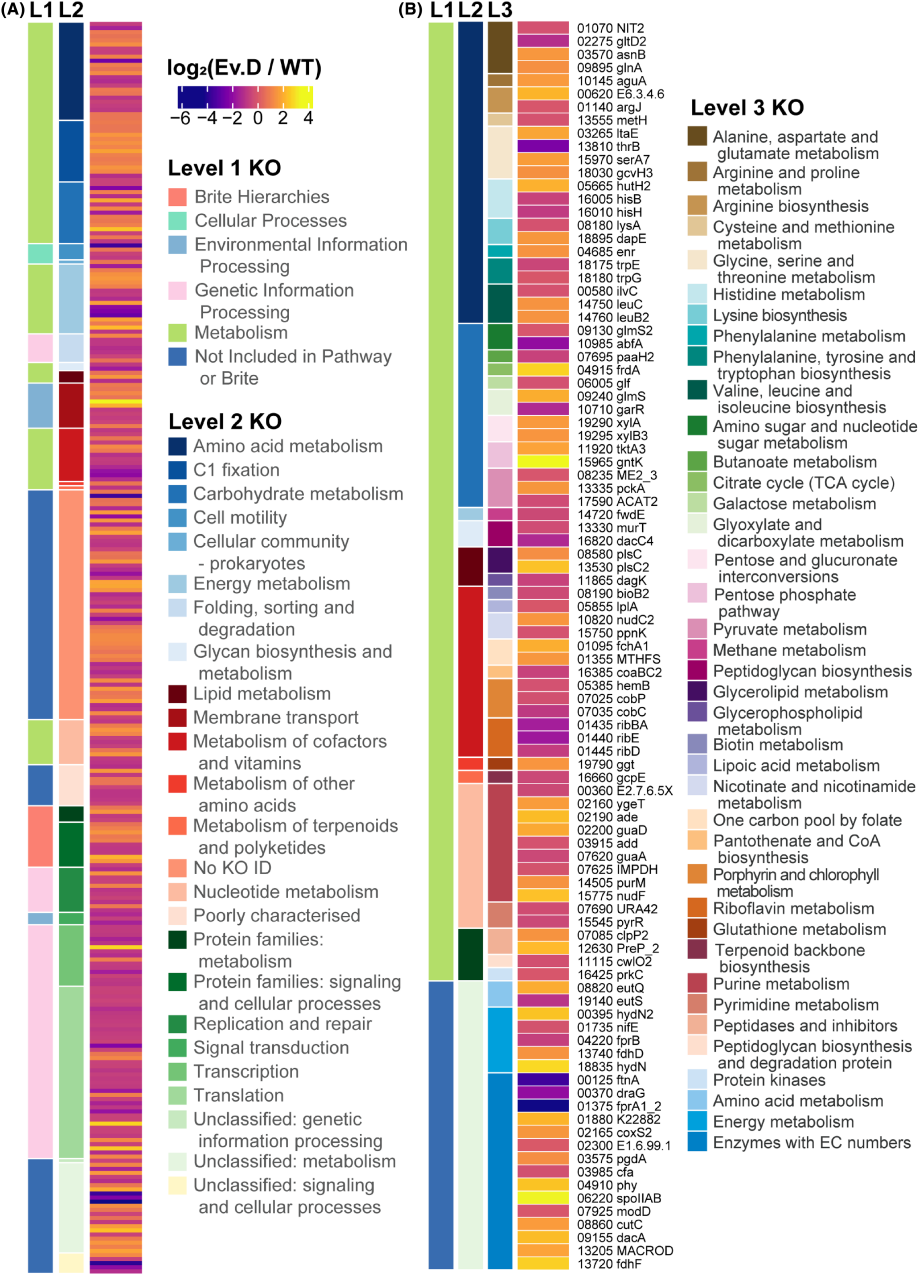
Changes to the proteome are widespread between Evolved D (Ev. D) and its parental strain (WT) under the same conditions. (A) The average log_2_(ratio) of 305 differential expressed proteins are mapped to KEGG Orthology (KO) Level 1 (L1) and Level 2 (L2) fields from Valgepea et al. (2022), sorted by alphabetized L2. (B) From the 305 proteins in (A), those directly linked to metabolism are shown with KO Level 3 (L3, except for those shown in Figure 4, total of 97 proteins), accession number (preceded by CAETHG_RS) and proposed protein name, sorted by accession number, L3 and L2.

**FIGURE 4:**
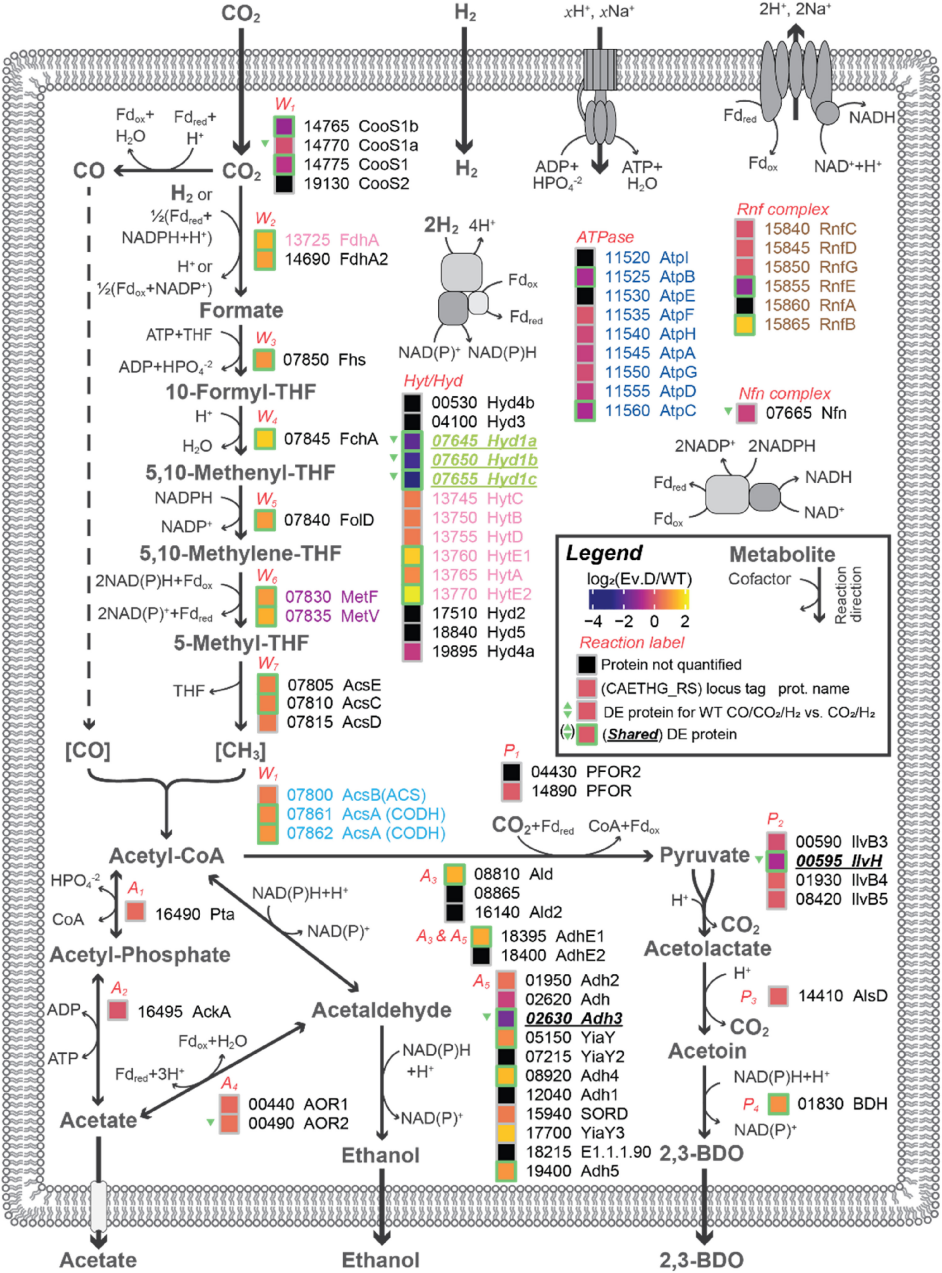
Protein expression changes for the main metabolic pathways of *Clostridium autoethanogenum*, with Evolved D (*n* = 3) and its parental strain (*n* = 3) fermenting CO_2_/H_2_ in chemostats. See legend for figure details. Differentially expressed (DE) proteins from comparison of DSM 19630 (WT) chemostat fermentations with CO/CO_2_/H_2_ (*n* = 2) and CO_2_/H_2_ (*n* = 3) are also shown by green triangles (pointing down indicates higher expression in CO_2_/H_2_). Proteins in complex with others are shown by the colour of their locus tag and protein name. Metabolite abbreviations: BDO, butanediol; CoA, coenzyme A; THF, tetrahydrofolate. Enzyme/reaction labels (red): W1 – carbon monoxide dehydrogenase (CODH), can be in complex with acetyl‐CoA synthase (ACS); W_2_ – formate dehydrogenase (Fdh [bottom], can be in complex with HytA‐E [top]); W3 – formate:THF ligase; W4 – 5,10‐methenyl‐THF 5‐hydrolase; W5 – methylene‐THF dehydrogenase; W6 – methylene‐THF reductase; A1 – phosphotransacetylase; A_2_ – acetate kinase; A3 – acetaldehyde dehydrogenase; A4 – acetaldehyde:ferredoxin oxidoreductase; A5 – alcohol dehydrogenase; P1 – pyruvate: ferredoxin oxidoreductase; P_2_ – acetolactate synthase; P3 – acetolactate decarboxylase; P4 – (R)‐2,3‐butanediol dehydrogenase; Hyt/Hyd – hydrogenase; ATPase – F0F1‐ATP synthase; Rnf complex – proton‐translocating ferredoxin:NAD+ oxidoreductase; Nfn complex – ferredoxin‐dependent transhydrogenase.

Comparative proteomic analysis of Evolved D against its parental strain from steady CO_2_/H_2_ chemostat fermentations revealed 371 differentially expressed (DE) proteins of 1713 proteins detected (see Figure S5A–C for a summary of the data). Of these, 305 had KEGG Orthology (KO) (from (Valgepea et al., 2022), Figure 3A), mapping predominantly to ‘Metabolism’ (48%) and ‘Genetic Information Processing’ (36%), excluding those ‘Not Included in Pathway or Brite’ or without mapping (i.e., ‘#N/A’, if summed equals 43% of total). While ‘Metabolism’ had relatively mixed changes, we observed that DE proteins related to ‘Genetic Information Processing’ generally had lower expression in Evolved D than its parental strain (Figure 3). Yet, a transcriptional machinery, translation factor and ribosome biogenesis protein (KO Level 3, CAETHG_RS06215, 09610 and 16665, log_2_FC of 2.8, 2.5 and 2.7) were amongst the proteins that change in expression strongly. Interestingly, proteins related to sugar metabolism were also amongst highly increased proteins in Evolved D, including a monosaccharide ABC transporter (CAETHG_RS06665) and a gluconate transporter and kinase (CAETHG_RS15960 and 15965) with log_2_FCs of 3.3, 2.0 and 2.3. Notable strongly decreased DE proteins in Evolved D included proteins related to metal‐mediated electron transfer (CAETHG_RS00120, 00125, 01375 and 07290; log_2_FCs of −3.7, −3.8, −4.4 and −4.0), fatty‐acid biosynthesis (CAETHG_RS00375, log_2_FC 4.4), methyl‐accepting chemotaxis (CAETHG_RS00365, log_2_FC −3.7) and carbon starvation signalling (CAETHG_RS07715, log_2_FCs −2.1). The homoserine kinase (CAETHG_RS13810, thrB) containing a single‐nucleotide variant (SNV) mutation (Table 1) was also present in highly decreased differentially expressed proteins (with −2.7 log_2_FC) – the only mutation also associated with a DE protein. Excluding the primary metabolic pathway (i.e., C1 fixation, energy metabolism and energy conservation), DE proteins directly involved in metabolism did not show any clear trends or changes describing increased metabolic fitness (Figure 3B). However, DE proteins in the central metabolic pathways did show trends. Compared to its parental strain, Evolved D had a significant increase in protein for at least one enzyme involved in every WLP reaction (Figure 4), except for acetyl‐CoA synthase (AcsB). However, specific isoenzymes diverged from this general trend. While the carbon monoxide dehydrogenase that is part of the WLP gene cluster (AcsA) increased in expression, the other quantified version (CooS1, CooS1a and CooS1b – expected to form a complex [Valgepea et al., 2022]) had decreased expression for two of its three proteins. A significant decrease in expression was also observed for hydrogenase Hyd1, while half (3/6) of hydrogenase HytA‐E significantly increased (the rest showing a significant increase if using a fold‐change threshold of ~1.4 or |log_2_FC| ≥ 0.5). Interestingly, both HytE proteins were amongst the highly increased DE proteins (top 25, log_2_FC = 1.7 and 2.2), while Hyd1 proteins were amongst the highly decreased DE proteins (top 20, log_2_FCs = −2.1, −2.4 and −2.9). Both formate dehydrogenases (FdhA and FdhA2) increased, where HytA‐E forms an NADP‐specific electron‐bifurcating [FeFe]‐hydrogenase in complex with FdhA (Wang et al., 2013). These proteins directly metabolize CO_2_ and H_2_, so changes to their expression are more likely to cause differences in robustness.

Of 58 DE proteins between DSM 19630 CO/CO_2_/H_2_ and CO_2_/H_2_ fermentations (see Figure S6A–C for a summary of the data), 29 are shared DE proteins with CO_2_/H_2_ fermentation by Evolved D and DSM 19630 (Table S6). A greater number of DE proteins for DSM 19630 CO/CO_2_/H_2_ versus CO_2_/H_2_ fermentations have reduced expression (62%, i.e., higher in CO_2_/H_2_). Shared DE proteins were also more likely to have greater expression in DSM 19630 CO_2_/H_2_ (−log_2_FC) for both comparisons (85%). Strongly decreased and shared DE proteins include Adh3, Hyd1[a,b,c], ThrB, CAETHG_RS00120, 00125, 01375 and 07290 (metal‐mediated electron transfer), CAETHG_RS00365 (methyl‐accepting chemotaxis) and CAETHG_RS07715 (carbon starvation signalling). CAETHG_RS07785, 08820, 08860 and 19150 (RNA binding, ethanolamine utilization, choline trimethylamine‐lyase and molybdopterin binding) are the only shared DE proteins with increased expression, CAETHG_RS07785 and 08820 just reaching the cut‐off threshold of ≥2 unique number of peptides for CO/CO_2_/H_2_ versus CO_2_/H_2_. Outside of thrB/ThrB as a mutated gene and a shared DE protein, these shared DE proteins may be more relevant to the metabolic changes between Evolved D and its parental strain than DE proteins from either comparison by itself.

### Alterations to intracellular metabolite profiles are non‐influential to metabolism

**FIGURE 5:**
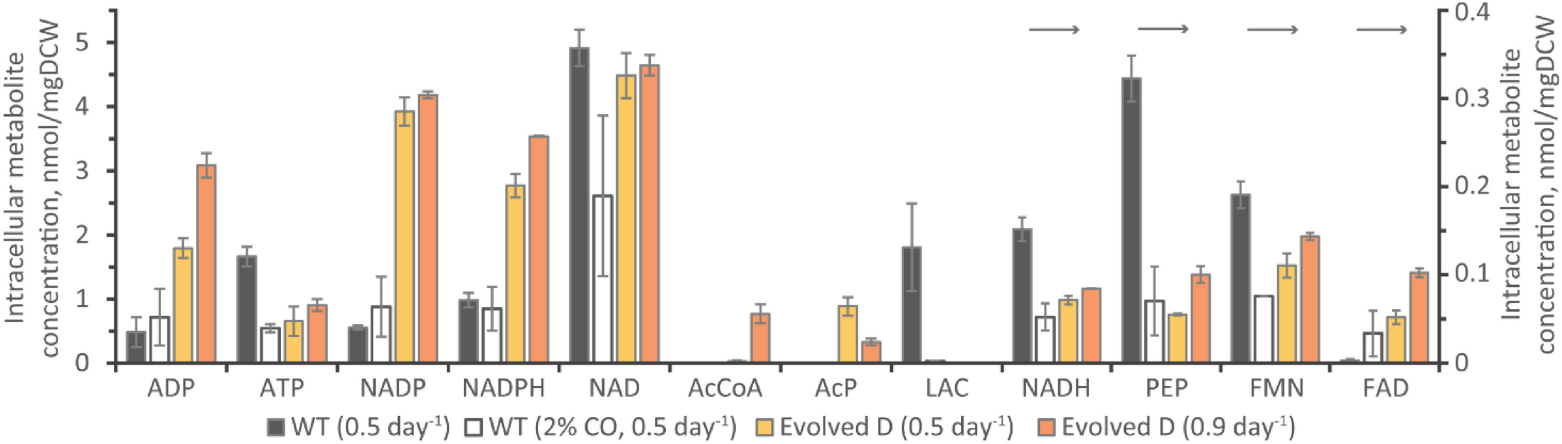
Quantification of key intracellular metabolites from *Clostridium autoethanogenum* DSM 19630 (WT) and Evolved D lineage. Metabolites are arranged by corresponding axis (indicated by arrows). Dilution rate of 0.5 day^−1^ was quantified for WT (*n* = 3) and Evolved D (*n* = 3) and 0.9 day^−1^ for Evolved D (*n* = 2). Results for WT grown with CO/CO_2_/H_2_ (2% CO, 0.5 day^−1^, *n* = 2) are also shown, while other conditions were grown on CO_2_/H_2_. Bars represent the average ±SD of bioreplicates. AcCoA, acetyl‐CoA; AcP, acetyl‐phosphate; ADP, adenosine diphosphate; ATP, adenosine triphosphate; FAD, flavin adenine dinucleotide; FMN, flavin mononucleotide; LAC, lactate; NAD(H), oxidized (reduced) nicotinamide adenine dinucleotide; NADP(H), oxidized (reduced) nicotinamide adenine dinucleotide phosphate; PEP, phosphoenolpyruvate.

Thermodynamic principles determine autotrophic acetogen metabolism (Mahamkali et al., 2020; Richter et al., 2016). Reaction feasibilities can control metabolism via intracellular metabolite concentration (e.g., redox or cofactors). Furthermore, proteome quantification has not solely explained changes to autotrophic metabolism previously, while metabolomics has explained changes to autotrophic metabolism (Heffernan et al., 2022). Only NAD concentration did not change between Evolved D and WT fermentation of CO_2_/H_2_, where Evolved D had increased ADP, NADP(H), acetyl‐phosphate and FAD concentrations (Figure 5). However, Evolved D had reductions to NADH, phosphoenolpyruvate (PEP), lactate and FMN concentrations. Although pyruvate was not quantified, reductions to PEP and lactate also suggest a reduction in pyruvate concentration for Evolved D. High NADPH/NADP ratios were seen during H_2_ utilization by *C. autoethanogenum* (~0.44) (Mahamkali et al., 2020). Although NADPH concentration increases for Evolved D by 2.82‐fold (±0.38, *p* = 0.0003), NADPH/NADP reduces by 0.40‐fold (±0.03, *p* = 0.0005). Furthermore, both NADH concentrations (0.47‐fold ±0.05, *p* = 0.001) and NADH/NAD ratios decreased by 0.47‐ and 0.51‐fold for Evolved D (±0.05, *p* = 0.001, and ±0.08, *p* = 0.01, respectively).

Interestingly, shifts to the intracellular metabolites did not lead to rate or titre changes between strains in the same condition, whereas metabolite changes were linked to acidogenesis and solventogenesis in *C. ljungdahlii* PETC (Richter et al., 2016). Higher NADP(H) was linked to acidogenesis, while higher NADH was associated with solventogenesis – in agreement with our results when increasing dilution rate. The switch to 0.9 day^−1^ and acidogenesis by Evolved D changed only NADPH (by 1.28‐fold ±0.02, *p* = 0.02) of the redox metabolites, where acetyl‐phosphate and acetyl‐CoA also show changes typical of a solventogenesis–acidogenesis shift (0.37‐fold ±0.09, *p* = 0.03, and 25‐fold ±10, *p* = 0.01, respectively) for syngas growth of WT (Valgepea, de Souza Pinto, et al., 2017). Thus, adapting Evolved D to faster growth may have led to higher NADP(H) concentrations, where only growth close to D_max_ led to acidogenesis (for both Evolved D and WT). This indicates that cells adapted to keeping their redox metabolism prepared for faster growth, but this did not influence the rates and titres at 0.5 day^−1^.

## DISCUSSION

Following our previous work with *C. autoethanogenum* fermenting CO_2_ in bioreactors (Heffernan et al., 2020), we believed that there was potential to use adaptive laboratory evolution to improve specific growth rate. Previous studies fermenting CO_2_ as the only carbon source and H_2_ as electron donor using *C. autoethanogenum* (Mock et al., 2015) and closely related *C. ljungdahlii* (Emerson et al., 2019; Klask et al., 2020; Molitor et al., 2019) suggested that it would be difficult for organisms to maintain the production of ethanol while improving specific growth rate. This is due to the differences in overall redox metabolism between acetate and ethanol production, where ethanol production has a Gibbs free energy of reaction (ΔᵣG^0^) of −105 kJ/mol and consumes 6H_2_, while acetate production has a ΔᵣG^0^ of −95 kJ/mol and consumes 4H_2_ (leading to 0.25 ATP/H_2_ compared to 0.20 ATP/H_2_ for ethanol). Furthermore, *Acetobacterium woodii*, an organism that can grow faster than either organism with CO_2_/H_2_ produces only acetate natively (Kantzow et al., 2015). As we knew supplementing CO could enhance CO_2_/H_2_ fermentation (Heffernan et al., 2020), ALE with CO/CO_2_/H_2_ attempted to offset redox limitations of CO_2_/H_2_ fermentation and encourage *C. autoethanogenum* to retain ethanol production.

If designed appropriately, ALE pressures do not necessarily have to be the target conditions or fixed – previously observed with *S. ovata* where methanol ALE also improved CO_2_ fermentation (Tremblay et al., 2015) and ALE of *S. cerevisiae* using alternating passages with and without methanol to improve methanol assimilation (Espinosa et al., 2020). Here, CO supplementation during ALE appeared to retain ethanol production during CO_2_/H_2_ fermentation (Figure 1B). All lineages from CO_2_/H_2_ ALE may also have retained ethanol production in chemostats, but they were only tested in bottle experiments. Evolved D and its parental strain had the same rates and titres under the same chemostat conditions and shifted to acetate‐only production when approaching their maximum specific growth rate (Figure 2D; Figure S3). A different ALE method may be required to improve ethanol production rates, titres or selectivity as early exponential phase passaging does not apply selective pressure for production. Growth at lower pH or in an environment with a tightly controlled pH is known to facilitate acetate reduction in *C. ljungdahlii* (Klask et al., 2020; Richter et al., 2016) and may be feasible for improving ethanol production.

Use of heterotrophic/mixotrophic growth for colony picking can lead to reduction/removal of selective pressure required for ALE, while picking gas fermenting ALE colonies is difficult for several reasons: (i) autotrophic growth on solid medium is very slow; (ii) anaerobic culturing has greater limitations in using high‐throughput methods (e.g., automatic colony picking and multi‐well plate culturing of single colonies require facilities like an anaerobic biofoundry [Fackler et al., 2021]), while low‐throughput methods could lose coverage of mutation data/information; and (iii) it is also challenging to maintain anaerobicity while using safe methods (i.e., minimizing risk when transitioning between cell growth and colony picking). Colony picking was not used here. While colony growth can lead to ‘mutational jackpot events’ and, therefore, genomic heterogeneity (Fusco et al., 2016) (i.e., encourage random mutagenesis), the significant changes to growth and proteome observed here show the validity of our ALE method. Furthermore, many of the differences observed at the proteome level were matched for DSM 19630 fermentations with CO_2_/H_2_ vs. CO/CO_2_/H_2_ (Table S6). Nonetheless, we believe that future ALE studies for CO_2_/H_2_ fermentation are needed to elucidate the metabolism of CO_2_/H_2_, particularly through the promotion of mutation events and use of high‐throughput screening and analysis methods.

Outside of *C. autoethanogenum*'s core metabolism, many gene functions and relationships are unknown (Valgepea et al., 2022). We show here that ALE studies can assist this understanding by pointing to genes that influence key metabolic aspects (e.g., redox homeostasis and metabolic regulation). Genomic analysis alone could not elucidate the changes leading to the improved phenotype. However, in combination with proteomics and metabolomics, we could elucidate the key functions leading to the enhanced phenotype. Proteomics revealed a range of DE proteins between Evolved D and its parental strains (Figure 3). We believe thrB is a notable gene as it was mutated in Evolved D (Table 1), a DE protein for CO_2_/H_2_ chemostat fermentation between Evolved D and WT (Figure 3) and a DE protein for WT chemostat fermentation with CO_2_/H_2_ and CO/CO_2_/H_2_ (Table S6). ThrB is involved in ‘glycine, serine and threonine metabolism’, a pathway not known to be important for autotrophic metabolism besides biomass generation (biomass being ~3% of carbon flux here). Serine and glycine did have lower intracellular concentrations when CO_2_/H_2_ growth of *C. ljungdahlii* was enhanced by balancing redox with nitrate supplementation (Emerson et al., 2019), but this should impact only a small fraction of carbon flux. Furthermore, growth with nitrate caused less expression of RNA transcripts relating to the WLP (the opposite of Evolved D) and energy‐conserving pathways, potentially due to decoupling ATP generation from the WLP.

Increased expression of the WLP appears to facilitate improved growth in chemostats (Figure 4). However, our results show that specific isoenzymes' expression changes are more important than others. Knockout of CooS1 in *C. autoethanogenum* DSM 10061 led to apparent growth enhancement on CO_2_/H_2_ previously (Liew et al., 2016). Here, CooS1 and CooS1b had lower DE in Evolved D, and some growth improvement may be directly attributed to this (CooS1, CooS1a and CooS1b are expected to form a complex [Valgepea et al., 2022]). Furthermore, CooS1a was also less expressed in WT fermentations with CO/CO_2_/H_2_ compared to CO_2_/H_2_ (log_2_FC = −1.47), while CooS1 and CooS1b decreased but not significantly (log_2_FC ≈ −0.65, *p*‐value ≈ 0.2, Table S6). *C. autoethanogenum* fermenting CO‐based gases had variable protein concentrations for CooS1, CooS1a and CooS1b (i.e., between 10 and 170 nmol/gDCW), syngas having higher concentrations than CO or CO/H_2_ (~1.5‐fold) (Valgepea et al., 2022). AcsA(CODH) enzymes also had variable concentrations (i.e., 5 and 50 nmol/gDCW for CAETHG_RS07861‐07862) but were consistent between gas types. Therefore, as CooS1 is believed to function separate to the essential autotrophic enzyme AcsA (ACS), changes to expression of CooS1 may be more viable for cells. Mutations to *E. limosum*'s neighbouring acsA and cooC2 genes were found when implementing ALE for growth with higher CO concentrations (Kang et al., 2020), where a SNV mutation to the metabolically functional gene (acsA) was not in the active site. Structural analysis shows the importance of channelling CO_2_ (and CO) inside *C. autoethanogenum*'s CODH/ACS (Lemaire & Wagner, 2021), hypothesized by Liew et al. (2016) to be highly important for growth with CO_2_/H_2_. Our findings agree with their hypothesis that CODHs appear to represent an important metabolic engineering strategy to improve gas utilization efficiency.

Decreased expression of Hyd1 and increased expression of HytA‐E suggest that HytA‐E is the more critical hydrogenase for CO_2_/H_2_ fermentation in *C. autoethanogenum* (Figure 4). All Hyd1 proteins were also less expressed between WT fermentations of CO/CO_2_/H_2_ and CO_2_/H_2_ (log_2_FC = −1.05, −1.35, −1.47, Table S6). Furthermore, H_2_ uptake was higher for Evolved D after the start of continuous culture (Figure 2C), suggesting that downstream reactions limit hydrogenase activity and HytA‐E could achieve even higher uptake rates. Interestingly, proteins linking HytA‐E with FdhA, HytE1 and HytE2 (Schuchmann et al., 2018), had the lowest concentration of the HytA‐E complex when cells were grown with CO‐based gases (Valgepea et al., 2022) and the highest increase in Evolved D. Further analysis of these characteristics and Hyt protein function within HytA‐E would generate meaningful engineering hypotheses.

Many isozymes of alcohol dehydrogenase are present in *C. autoethanogenum*'s proteome during CO_2_/H_2_ fermentation (Figure 4). Mock et al. (2015) suggest that acetaldehyde reduction to ethanol is more favourable with NADH for CO_2_/H_2_ fermentation. Here, we found two NADPH‐dependent alcohol dehydrogenases were DE proteins: Adh3 had lower expression after ALE, while Adh4 had higher. Other DE proteins capable of converting acetaldehyde to ethanol include AdhE1, Adh5 and YiaY – all of which had increased expression and have unknown cofactor dependencies. Potentially, one of these is functional with NADH, but not expressed by DSM 10061 (Mock et al., 2015). Otherwise, the only known NADH‐dependent alcohol dehydrogenase of *C. autoethanogenum*, Adh2, had only a slight increase in expression (non‐DE due to log_2_FC = 0.39, but *q*‐value = 0.0014). Furthermore, the capability to use either NADPH or NADH for ethanol production may be advantageous for robustness, depending on specific cell conditions at a given moment.

CO_2_/H_2_ fermentation to liquid products has significant potential for sustainable biomanufacturing. Almeida Benalcázar et al. (2022) recently showed this potential by modelling a large‐scale ethanol production process, showing ideal fermentation conditions could facilitate improved feasibility. These would realize a similar ethanol cost and a lower CO_2_ abatement cost than first‐ and second‐generation ethanol while also lowering the global warming potential. Achieving idealized conditions would circumvent current limitations associated with renewable CO_2_/H_2_ valorization (Almeida Benalcázar et al., 2022; Wood et al., 2021) through selective ethanol production to a high‐titre and optimized gas–liquid mass transfer properties. Furthermore, combinations of C1‐based processes may be able to offset beneficial attributes of the individual processes. For example, co‐utilization of CO and CO_2_ (with H_2_) can improve CO metabolism's energy efficiency (Simpson & Köpke, 2020) and CO_2_ metabolism's titres (Heffernan et al., 2020; Novak et al., 2021). The ability to produce more valuable products may also help this endeavour, where optimized acetone and isopropanol production pathways were recently shown at large scale (Liew et al., 2022). Research on CO_2_ fermentation can leverage some knowledge from CO‐based fermentations, but improving our understanding of CO_2_‐based processes may also be essential.

## CONCLUSION

Various methods of ALE achieved improved CO_2_/H_2_ growth by *C. autoethanogenum*, where we obtained a strain via ALE with CO_2_/H_2_ supplemented with 2% CO (Evolved D) with an optimum trade‐off between improving growth and maintaining ethanol production. Comparison of Evolved D in chemostats shows that the lineage performs similarly to its parental strain, but can grow faster in certain conditions. Phenome, proteome and metabolome analyses of Evolved D and its parental strain thoroughly characterize CO_2_/H_2_ metabolism, the most complete quantification to date. We subsequently show that multi‐omics strain characterization is useful to elucidate novel and industrially relevant cellular mechanisms in acetogens.

## AUTHOR CONTRIBUTIONS

**James Heffernan:** Conceptualization; data curation; formal analysis; investigation; methodology; software; validation; visualization; writing – original draft; writing – review and editing. **R. Axayactl Garcia Gonzalez:** Methodology; resources; supervision. **Vishnu Mahamkali:** Investigation. **Tim McCubbin:** Software; validation; writing – review and editing. **Dara Daygon:** Investigation; methodology. **Lian Liu:** Investigation; methodology. **Robin Palfreyman:** Software; validation. **Audrey Harris:** Methodology; supervision. **Michael Koepke:** Methodology; supervision. **Kaspar Valgepea:** Supervision; writing – review and editing. **Lars Keld Nielsen:** Supervision; writing – review and editing. **Esteban Marcellin:** Conceptualization; funding acquisition; methodology; project administration; resources; supervision; validation; writing – review and editing.

## FUNDING INFORMATION

Novo Nordisk Foundation (Grant/Award Number: NNF20CC0035580 and NNF14OC0009473). Queensland Government. Estonian Research Council's grant agreement PSG289. Australian Research Council (Grant/Award Number: CE200100029). European Union's Horizon 2020 research and innovation programme (Grant/Award Number: N810755).

## CONFLICT OF INTEREST STATEMENT

LanzaTech has interest in commercial application of gas fermentation with *C. autoethanogenum*. VM, AH and MK are employees of LanzaTech.

## Supporting information


Figures S1–S6.

Tables S1–S6.

Appendices S1–S3.
