# Supplementary material for: Adaptive laboratory evolution of Clostridium autoethanogenum to metabolize CO2 and H2 enhances growth rates in chemostat and unravels proteome and metabolome alterations
Source: Microb Biotechnol. 2024 Apr 3;17(4):e14452. doi: 10.1111/1751-7915.14452 (PMC10990044; doi:10.1111/1751-7915.14452)
Supplement: Supplementary file 1 — Figures S1–S6. [file MBT2-17-e14452-s001.docx]

Supporting file for

Lab-evolution of *Clostridium autoethanogenum* modifies proteome of CO₂/H₂ chemostat fermentation while metabolites control phenotype.

James Heffernan^a,b^, Ricardo A. Garcia Gonzalez^a,b^, Vishnu Mahamkali^c^, Tim McCubbin^d^, Dara Daygon^d^, Lian Liu^d^, Robin Palfreyman^d^ Audrey Harris^c^, Michael Koepke^c^, Kaspar Valgepea^f^, Lars Nielsen^a,b,d,e^, Esteban Marcellin^a,b,d,*^

^a^ Australian Institute of Bioengineering and Nanotechnology, The University of Queensland, Corner Cooper Rd & College Rd, St. Lucia, QLD 4072, Australia. ^b^ ARC Centre of Excellence in Synthetic Biology, The University of Queensland, St. Lucia, QLD 4072, Australia. ^c^ LanzaTech Inc., Skokie IL 60077, United States. ^d^ Queensland Metabolomics and Proteomics Q-MAP, The University of Queensland, St. Lucia QLD 4072, Australia. ^e^ The Novo Nordisk Foundation Center for Biosustainability, Technical University of Denmark, DK-2800 Kgs. Lyngby, Denmark. ^f^ ERA Chair in Gas Fermentation Technologies, Institute of Technology, University of Tartu, 50411 Tartu, Estonia.

Contents

[1. Supporting figures S1-S6 2](#_Toc155195728)

# Supporting figures S1-S6


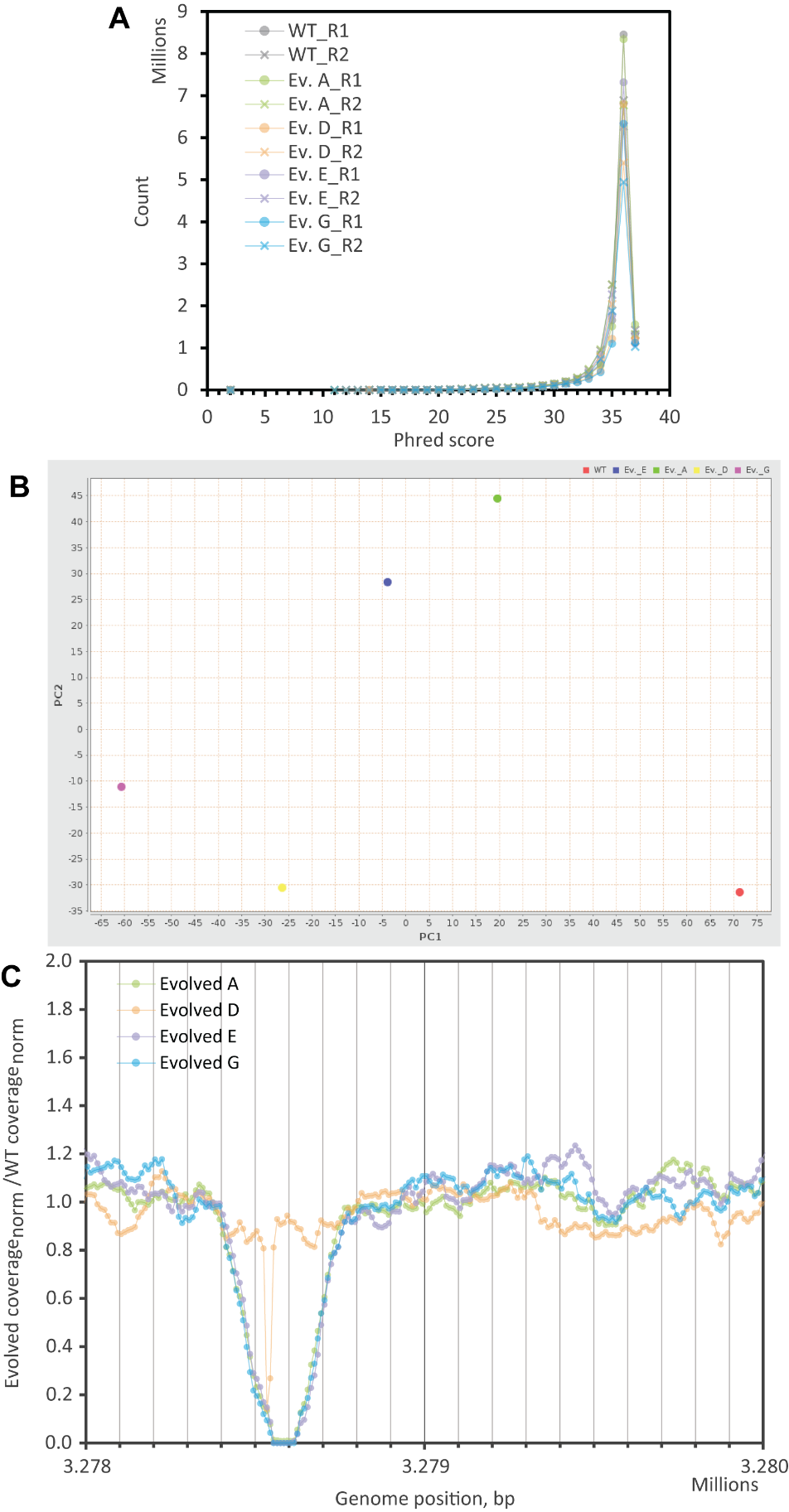
**Figure S1.** Sequencing quality and selected alignment data of Evolved lineages (Ev. A, D, E, G) and their parental strain (WT). (**A**) Analysis of raw read quality by FastQC (https://www. bioinformatics.babraham.ac. uk/projects/fastqc/) showing high quality sequencing for both forward (R1) and reverse (R2) strands (i.e., Phred of 30 = base call accuracy of 99.9%, and 40 = 99.99%). Data points represent the number of points within an integer Phred score bin. (**B**) PCA of sequenced lineages from multi-sample BamQC of sequence alignments by snippy. Appropriate separation between lineages is shown with slight condition-dependent grouping (i.e., by ALE gas). (**C**) Deleterious mutation of a large section of genome (at ~3.2785 Mb) was identified using the per-base coverage output from QualiMap BamQC analysis of sequence alignments. Per-base coverage was normalized ($\mathrm{coverag}e_{\mathrm{norm}}$) by the overall mean coverage of its alignment (560, 543, 471, 516 and 448 for WT, Evolved A, D, E and G respectively) first, then Evolved lineages were normalized by WT. Data points represent the mean of 10 bp. Evolved/WT $\mathrm{coverag}e_{\mathrm{norm}}$ drops below 25% (= 0.25) at ~150/456 bp into *argR*, see **Table** **S3** for mutation details.

**
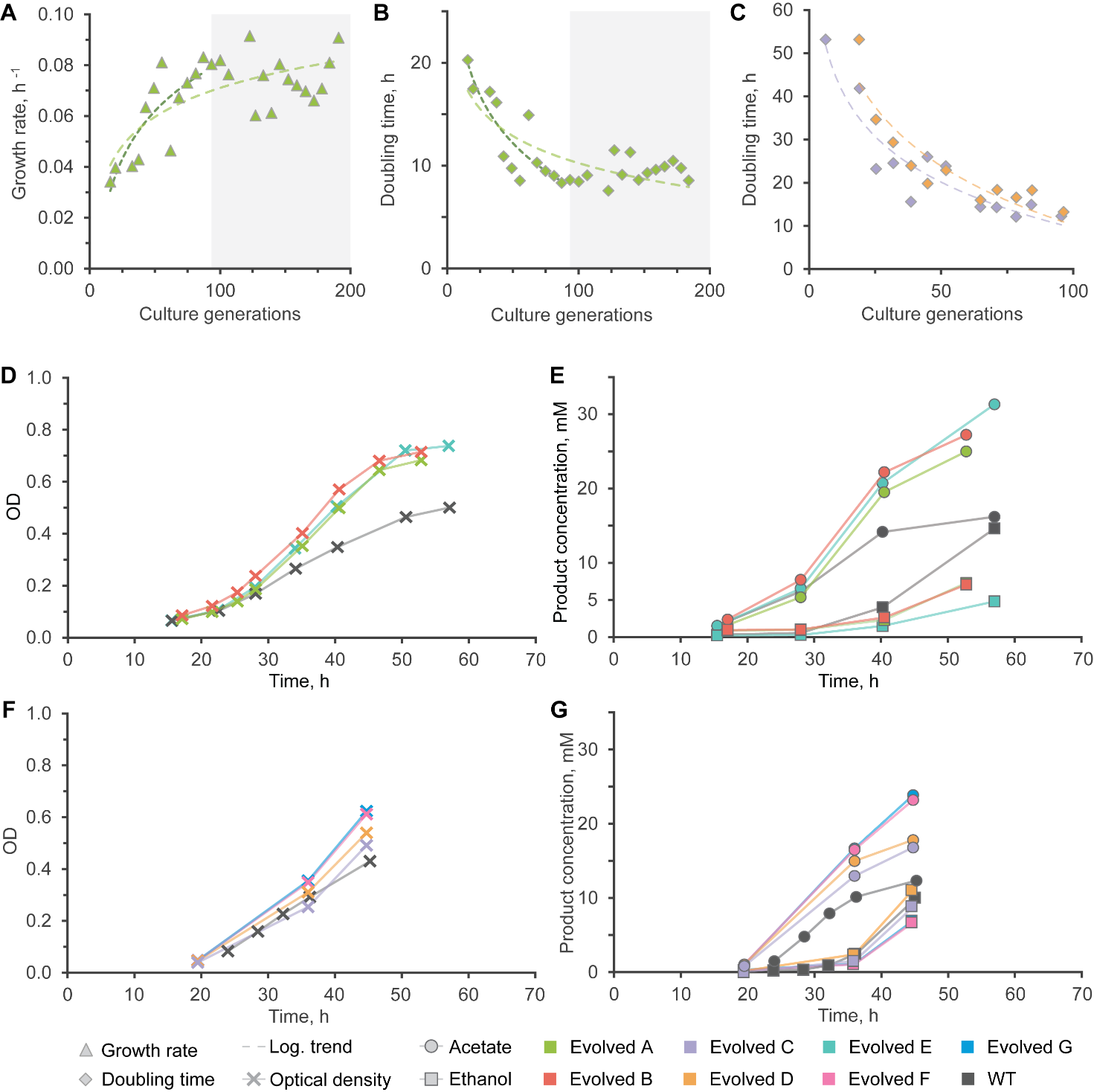
**

**Figure S2.** ALE of C. autoethanogenum for improved growth with CO₂/H₂. (**A**) Typical profile of changes to maximum growth rate during ALE, showing Evolved A here. Grey area shows culture generations where no or minimal improvements to growth rate were observed (after ~90 h). Logarithmic trends are fitted to indicate asymptotic approach to (local) minima and emphasize lack of change after 90 h. (**B**) Minimum doubling time matches maximum growth rate from (**A**), to demonstrate harshness and variability of maximum growth rate compared to minimum doubling time. Minimum doubling time was an easier experimental parameter to base ALE criteria on (e.g., termination and bottles to glycerol stock). (**C**) Change in minimum doubling time for Evolved C & D during ALE culturing. (**D-G**) Initial batch CO₂/H₂ growth and production of *C. autoethanogenum* ALE lineages from early to late exponential phase. Acetate and ethanol concentrations, and OD measurements represent individual cultures performed in 1 L Schott bottles (200 mL PETC-MES) pressurized with ~140 kPa CO₂/H₂ (otherwise same as 2.2 Bottle evolution). Due to the timing of ALE strain development and laboratory limitations, experiments with Evolved A, B & E (**D** & **E**) and Evolved C, D, F & G (**F** & **G**) were performed at different times/with different batches of medium, which may lead to some differences in growth – WT curves are performed for each instance to provide normalization. (**D**) Maximum growth rates were 0.101 h^-1^ (R² 1.00), 0.106 h^-1^ (R² 1.00) and 0.102 h^-1^ (R² 1.00) for Evolved A, B & E, and 0.084 (R² 1.00) for DSM 19630 (WT). (**F**) Maximum growth rates were 0.102 h^-1^ (R² 0.98), 0.099 h^-1^ (R² 0.96), 0.104 h^‑1^ (R² 0.96) and 0.104 h^-1^ (R² 0.96) for Evolved C, D, F & G, and 0.078 h^-1^ (R² 0.99) for WT. Both maximum growth rates for WT are within range of **Figure S4** (where n = 3, 0.081 ±0.005 h^-1^).


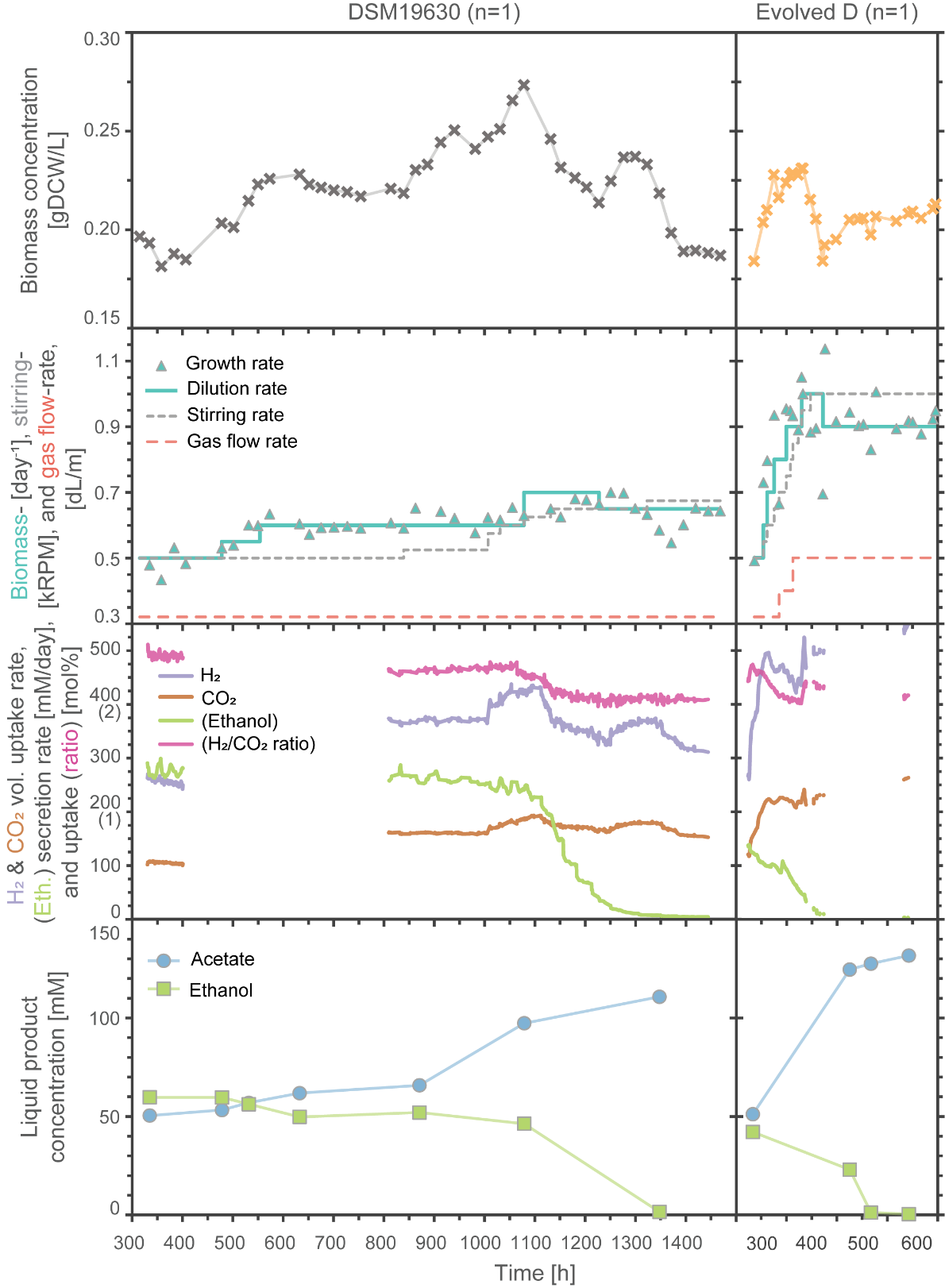


**Figure S3.** Assessing Evolved D robustness via increasing dilution rate. DSM19630 was grown with increasing dilution rate following our previous characterization of its growth with CO₂/H₂ [1]. Biomass concentration (gDCW/L – g-dry-cell-weight/L) and gas uptake (mM/day) data were used to assess if bioreactor conditions could be changed (i.e., increasing dilution rate or stirring), where conditions not presented here remained the same as described in 2.3 Chemostat testing (i.e., temperature and pH). Evolved D bioreactor conditions could be changed more rapidly based on these factors, and two replicates achieved steady state at 0.9 day^-1^, however, one is shown here for clarity. ‘Biomass’ rates compared set dilution rate and observed growth rate, which is calculated using the change in biomass concentration. Stirring speed (kRPM – kilo-RPM) and gas flow rate (dL/m – deci-L/minute) determine the gas-liquid mass transfer. Increasing these allows both strains to grow steadily at higher dilution rates. Gas uptake and production rates (mM/day) have higher resolution (/frequency) than manual measurements and show relationships between parameters and phenotypes. H₂/CO₂ uptake ratio (mol%) links to ethanol production, where 2.5 can maintain production and 2.0 loses production for both WT and Evolved D. H₂/CO₂ and ethanol use scale with values in brackets. Liquid ethanol concentrations (mM) agree with the relationship from gas data – decreasing to 0 mM for both strains at dilution rates close to their respective maximum growth rates, while acetate roughly doubles. See **Table S4** for further steady-state data and comparisons of Evolved D and its parental strain.


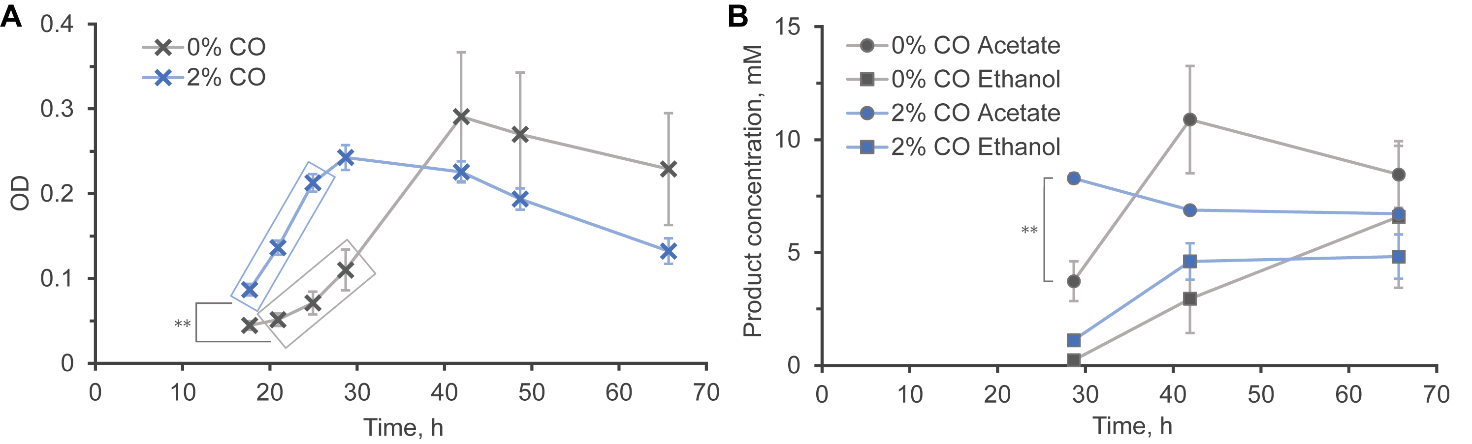
**Figure S4.** Batch growth with CO₂/H₂ and CO/CO₂/H₂. (**A**) Growth comparison shows the increase in maximum growth rate between CO₂/H₂ (0% CO) and CO/CO₂/H₂ (2% CO). (**B**) Minimal significant changes to the product concentration profiles where observed, only at ~30 h was CO/CO₂/H₂ acetate ~2.2-fold CO₂/H₂ (p-value = 0.014). The maximum growth rates (µ) are calculated from data in boxes and are 0.081 ±0.005 h^-1^ and 0.123 ±0.004 h^-1^ (p-value = 0.005). Acetate and ethanol concentrations, and OD measurements represent the mean ±standard deviation (SD) of three biological replicates. Cultivations were conducted using the same methods as 5.3.2 Bottle evolution, but without yeast extract.


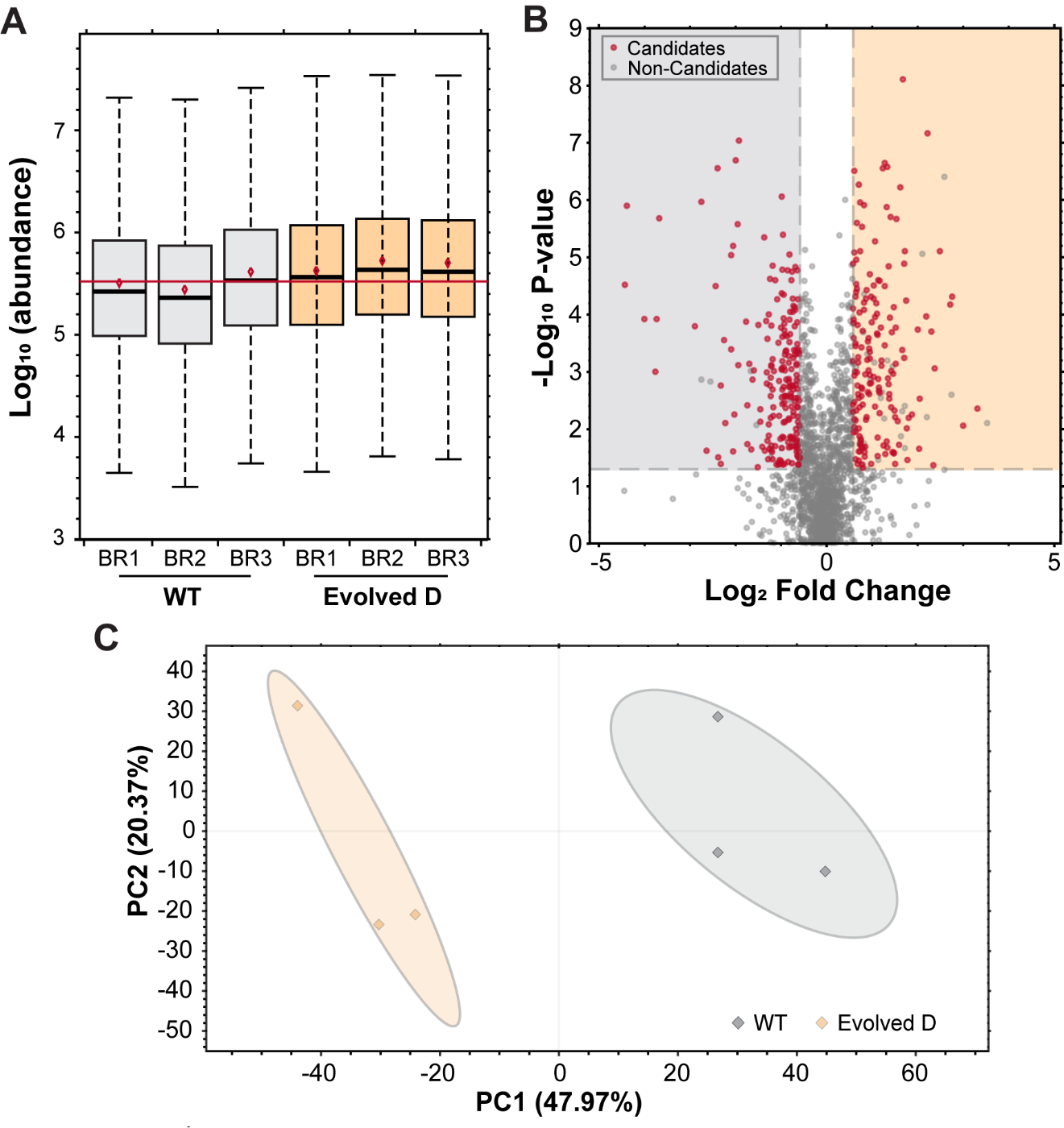


**Figure S5.** Summary of WT (grey) and Evolved D (orange) comparative proteomics via DIA mass spectrometry. (A) Protein abundances pre-normalization, showing high quality raw data. (B) Volcano plot showing differentially expressed proteins (candidates) and their thresholds - log₂FC ≥ 0.585, p-value ≤ 0.05, false discovery rate (FDR or q-value ≤ 0.01), and number of unique peptides ≥ 2. Colors indicate condition with higher expression (i.e., negative FC means higher expression in WT). (C) PCA showing good grouping within groups and separation between groups.


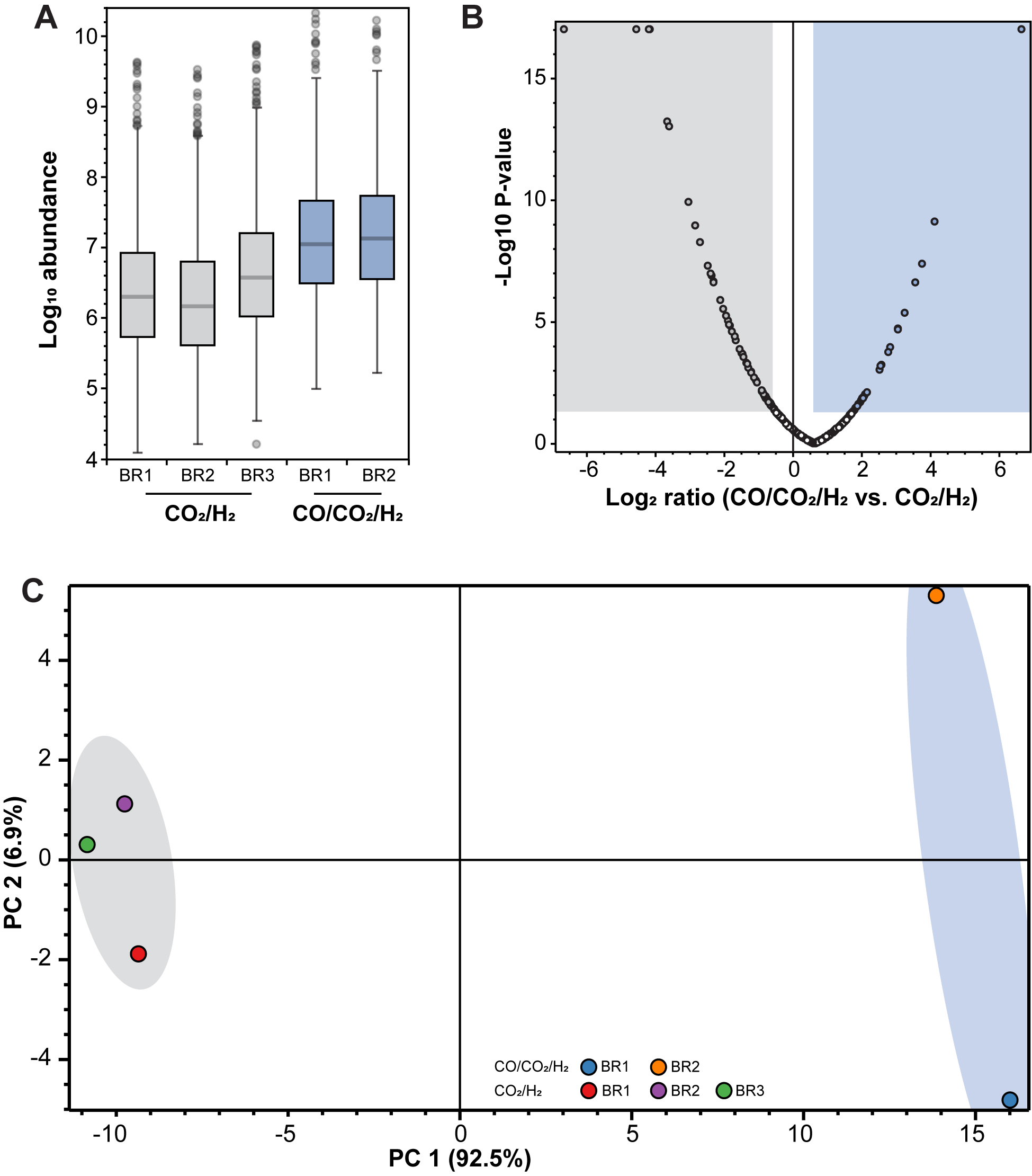


**Figure S6.** Summary of WT CO₂/H₂ (grey) and CO/ CO₂/H₂ (blue) comparative proteomics analysis via DDA mass spectrometry. between chemostats. These chemostat were previously cultured at dilution rate 0.50 day^-1^, described in [1]. (A) Protein abundances pre-normalization, showing good quality raw data. (B) Volcano plot showing differentially expressed proteins (candidates) and their thresholds - log₂FC ≥ 0.585, p-value ≤ 0.05, false discovery rate (FDR or q-value ≤ 0.01), and number of unique peptides ≥ 2. Colors indicate condition with higher expression (i.e., negative FC means higher expression in CO₂/H₂). (C) PCA showing good grouping within groups and separation between groups.

**References**

[1] J.K. Heffernan, K. Valgepea, R. de Souza Pinto Lemgruber, I. Casini, M. Plan, R. Tappel, S.D. Simpson, M. Köpke, L.K. Nielsen, E. Marcellin, Enhancing CO₂ -valorization using *Clostridium autoethanogenum* for sustainable fuel and chemicals production, Front. Bioeng. Biotechnol. 8 (2020) 204. https://doi.org/10.3389/fbioe.2020.00204.
