# Supplementary material for: Adaptive laboratory evolution of Clostridium autoethanogenum to metabolize CO2 and H2 enhances growth rates in chemostat and unravels proteome and metabolome alterations
Source: Microb Biotechnol. 2024 Apr 3;17(4):e14452. doi: 10.1111/1751-7915.14452 (PMC10990044; doi:10.1111/1751-7915.14452)
Supplement: Supplementary file 2 — Tables S1–S6. [file MBT2-17-e14452-s002.zip › mbt214452-sup-0003-TablesS2-S6.docx]

Supporting file for

Lab-evolution of *Clostridium autoethanogenum* modifies proteome of CO₂/H₂ chemostat fermentation while metabolites control phenotype.

James Heffernan^a,b^, Ricardo A. Garcia Gonzalez^a,b^, Vishnu Mahamkali^c^, Tim McCubbin^d^, Dara Daygon^d^, Lian Liu^d^, Robin Palfreyman^d^ Audrey Harris^c^, Michael Koepke^c^, Kaspar Valgepea^f^, Lars Nielsen^a,b,d,e^, Esteban Marcellin^a,b,d,*^

^a^ Australian Institute of Bioengineering and Nanotechnology, The University of Queensland, Corner Cooper Rd & College Rd, St. Lucia, QLD 4072, Australia. ^b^ ARC Centre of Excellence in Synthetic Biology, The University of Queensland, St. Lucia, QLD 4072, Australia. ^c^ LanzaTech Inc., Skokie IL 60077, United States. ^d^ Queensland Metabolomics and Proteomics Q-MAP, The University of Queensland, St. Lucia QLD 4072, Australia. ^e^ The Novo Nordisk Foundation Center for Biosustainability, Technical University of Denmark, DK-2800 Kgs. Lyngby, Denmark. ^f^ ERA Chair in Gas Fermentation Technologies, Institute of Technology, University of Tartu, 50411 Tartu, Estonia.

Contents

[1. Supporting Tables S2-S8 2](#_Toc155196163)

# Supporting Tables S2-S8

**Table S2**. Details of SNVs detected in each Evolved lineage.

| **ALE lineage** | **Mutation position** | **Mutation type, position and change to gene (DNA) [AA]^a^** | **Gene^b^, locus #^c^, and direction^d^** | **Gene description** |
| --- | --- | --- | --- | --- |
| Evolved A   - Chemostat CO_2_/H_2_ - Batch CO_2_/H_2_ | 1,565,629  (**E**, **G**) | missense snp, (256/423: C $\to$ A), [86/140: His $\to$ Asn] | *perR*, 07040, F | transcriptional repressor |
|  |  | - downstream variant (+228) | -, 07045, R | extracellular solute binding protein |
|  | 3,278,628  (**G**) | missense complex, (271/456: AATTT $\to$ TATTC), [91/151: AsnPhe $\to$ TyrSer] | *argR*, 14845, F | arginine repressor |
|  |  | - upstream variant (-496) | *TC.APA7*, 14850, F | amino acid permease |
|  |  | - downstream variant (+326) | *recJ2*, 14840, F | single-stranded-DNA-specific exonuclease |
| Evolved D   - Batch CO/CO_2_/H_2_ | 710,848 | non-coding snp (C $\to$ A) | - | - |
|  |  | - upstream variant (+115) | *gutA*, 03080, R | MFS transporter |
|  |  | - downstream variant (-51) | -, 03085, R | alcohol dehydrogenase catalytic domain |
|  | 1,606,333 | missense snp, (1537/1578: C $\to$ A), [513/525: Gln $\to$ Lys] | *gerKA*, 07225, F | spore germination protein |
|  |  | - upstream variant (-48) | *gerKC*, 07230, F | Ger(x) family spore germination protein |
|  | 2,526,452 | missense snp, (974/1107: G $\to$ T), [325/368: Arg $\to$ Ile] | ***prfB***, 11655, F | peptide chain release factor 2 |
|  |  | - upstream variant (-213) | -, 11660, F | MFS transporter |
|  | 3,047,148 | missense snp, (791/900: A $\to$ G), [264/299: Asn $\to$ Ser] | *thrB*, 13810, F | homoserine kinase |
|  |  | - upstream variant (-278) | -, 13815, F | GNAT-family N-acetyltransferase |
| Evolved E   - Chemostat D_max_ test CO_2_/H_2_ | 1,565,578  (**G**) | missense snp, (205/423: G $\to$ A), [69/140: Glu $\to$ Lys] | *perR*, 07040, F | transcriptional repressor |
|  |  | - downstream variant (+279) | -, 07045, R | extracellular solute binding protein |
|  | 1,565,629  (**A**, **G**) | missense snp, (256/423: C $\to$ A), [86/140: His $\to$ Asn] | *perR*, 07040, F | transcriptional repressor |
|  |  | - downstream variant (+228) | -, 07045, R | extracellular solute binding protein |
|  | 2,525,941  (**G**) | stop gained snp (463/1107: A $\to$ T) [155/368: Arg $\to$ *] | ***prfB***, 11655, F | peptide chain release factor 2 |
|  |  | - downstream variant (+484) | secA, 11650, F | preprotein translocase subunit |
| Evolved G   - Chemostat CO_2_/H_2_ - Batch CO/CO_2_/H_2_ | 1,565,578  (**E**) | missense snp, (205/423: G $\to$ A), [69/140: Glu $\to$ Lys] | *perR*, 07040, F | transcriptional repressor |
|  |  | - downstream variant (+279) | -, 07045, R | extracellular solute binding protein |
|  | 1,565,629  (**A**, **E**) | missense snp, (256/423: C $\to$ A), [86/140: His $\to$ Asn] | *perR*, 07040, F | transcriptional repressor |
|  |  | - downstream variant (+228) | -, 07045, R | extracellular solute binding protein |
|  | 2,525,941  (**E**) | stop gained snp (463/1107: A $\to$ T) [155/368: Arg $\to$ *] | ***prfB***, 11655, F | peptide chain release factor 2 |
|  |  | - downstream variant (+484) | *secA*, 11650, F | preprotein translocase subunit |
|  | 3,278,628  (**A**) | missense complex, (271/456: AATTT $\to$ TATTC), [91/151: AsnPhe $\to$ TyrSer] | *argR*, 14845, F | arginine repressor |
|  |  | - upstream variant (-496) | *TC.APA7*, 14850, F | amino acid permease |
|  |  | - downstream variant (+326) | *recJ2*, 14840, F | single-stranded-DNA-specific exonuclease |

^a^ Italicized gene variants indicate genes that start or end within 500 base pairs of the mutation, ^b^ gene names from Valgepea et al. [1], ^c^ locus number preceded by “CAETHG_RS”, ^d^ forward (F) and reverse (R) indicate gene direction. Conserved genome variants are indicated by bold letter of ALE lineage in ‘Mutation position’, conserved genome variants are indicated by bolded gene name (when not a conserved genome variant)

**Table S3.** DNA and amino acid changes for protein coding variants.

| **Gene** | **Ev.** | **Sequence Wild type:** dna **\|** AA **Variant: DNA \| aa** |
| --- | --- | --- |
| **07040**  ***perR*** | **A** | 256/423: C $\to$ A  1 atggataatttaacttctatttttagagaaaaaaagctaaaacttaccccacagcgtattgctgtatataaatatttacaatctaccaagaagcatccttcggttgaaaccatatataaagcccttcagctagaatatcctactatgagc 150 151 ctagcaactgtttataaggctctaaaaactttagttgatgtaaatttagtacaggaaatcaatataggagaaagcaactttagatatgatggaaatgttcatcct**A**attctcatatacagtgtatagtttgtgagaaagtagatgacgta 300 301 gaaggaatttgtttttctaatttaaatgataaaattaaagattgtgttgactacgaagtattaagtaatcaagtttatttttacggcatatgtaaagactgccaaaaaaattctaaagaataa 423  86/140: His $\to$ Asn (225)  1 MDNLTSIFREKKLKLTPQRIAVYKYLQSTKKHPSVETIYKALQLEYPTMSLATVYKALKTLVDVNLVQEINIGESNFRYDGNVHP**n**SHIQCIVCEKVDDVEGICFSNLNDKIKDCVDYEVLSNQVYFYGICKDCQ* 135 |
|  | **E G** | 205/423: G $\to$ A & 256/423: C $\to$ A  1 atggataatttaacttctatttttagagaaaaaaagctaaaacttaccccacagcgtattgctgtatataaatatttacaatctaccaagaagcatccttcggttgaaaccatatataaagcccttcagctagaatatcctactatgagc 150 151 ctagcaactgtttataaggctctaaaaactttagttgatgtaaatttagtacag**A**aaatcaatataggagaaagcaactttagatatgatggaaatgttcatcct**A**attctcatatacagtgtatagtttgtgagaaagtagatgacgta 300 301 gaaggaatttgtttttctaatttaaatgataaaattaaagattgtgttgactacgaagtattaagtaatcaagtttatttttacggcatatgtaaagactgccaaaaaaattctaaagaataa 423  69/140: Glu $\to$ Lys & 86/140: His $\to$ Asn (0.354 & 225)  1 MDNLTSIFREKKLKLTPQRIAVYKYLQSTKKHPSVETIYKALQLEYPTMSLATVYKALKTLVDVNLVQ**k**INIGESNFRYDGNVHP**n**SHIQCIVCEKVDDVEGICFSNLNDKIKDCVDYEVLSNQVYFYGICKDCQ* 135 |
| **07225**  ***gerKA*** | **D** | 1537/1578: C $\to$ A  1 gtgaagaagttttttaattctaaaaacacttcagatcttatttatgtacaaaagttatccgatgatagatataatttgcccttaaataaatctctttcaaataatttaaacgtattgcacgaattattttcaaattgtgctgatgttgtt 150 151 taccataaatttgttatacattcaatgaaatgttcctgcgtacttatttttataaatggactttcggatataagatcaattaatgaaagcatattaccttctatcatgaatattaaagaggttactgaaagtgatttaaaatgtgatcat 300 301 actgtagaaataattaaagaatattttcttgaaattgcaaaaataagtgaagtatcaactattgggcaaattacaaatgctctcctaaatggaaataccatacttttactagatagtgatgatatcgctttagaaatagaaacacccgga 450 451 tggaaggaaaaaagcgtatcagacacagatgttgaaaaagttataaggggaccaaatgaaggatttacgcaaaatatttccaccaatatatctcaactcagaagaaaaattaaatcttcagagctaaaatttgaagattttacagtaggg 600 601 aaacagacacaaacaaaaataagcattacctatttgcagggaatagtagataacaatattgttgaagatgtaaaaaagagactttccaaaatagacatagattctgttttagaaagtggatatatagaagaactgatagaagatacacac 750 751 tacacattatttccccaaattcagcacagcgaaaggccagatagagttgccgctgggattttagaaggtaggatagcactcttagtagatggtactccttgtgttttaatacttcctgctactttaattcaatttttgcaaactagtgag 900 901 gattattatgaaagatataccactaccatatttgtgcgtttcataaggttaattttcttcgtaatttcactgctgcttcctggatgttttgttgcaattattttatatcataaagaaatgattcctacacctttacttattagcattatg 1050 1051 ggagctgcccatggagttccttttcctatttttattgaagcactgttaatggaaacagcttttgaagcacttagagaagcaggcatccgtcttccttcaccagctaaccaaactgtaggcattgttggtgcccttgtcataggtgatgca 1200 1201 gccgttagagctggtgttatctctcctattatggttattgttatagccattacagctatagcttcttttagcattccatcttatgatatgggctatgccattcgtattttacgtttttccatgctgtgtttaggagcgtttttaggctta 1350 1351 tatggagttctgttaggtattattgtacttttaattcacttatcatcattaagctcctttggtgtaaattatctttctccactagcacctttaaatttaaaggaactcaaagacattttaataagattcccctggcctcatatgaaatgt 1500 1501 aggcctcattttgccaataataataatttgcaccgg**A**aaaaatcatcctatgaaaatggaaaagacgagaaaggataa 1578  513/525: Gln $\to$ Lys (0.219)  1 MKKFFNSKNTSDLIYVQKLSDDRYNLPLNKSLSNNLNVLHELFSNCADVVYHKFVIHSMKCSCVLIFINGLSDIRSINESILPSIMNIKEVTESDLKCDHTVEIIKEYFLEIAKISEVSTIGQITNALLNGNTILLLDSDDIALEIETPG 150 151 WKEKSVSDTDVEKVIRGPNEGFTQNISTNISQLRRKIKSSELKFEDFTVGKQTQTKISITYLQGIVDNNIVEDVKKRLSKIDIDSVLESGYIEELIEDTHYTLFPQIQHSERPDRVAAGILEGRIALLVDGTPCVLILPATLIQFLQTSE 300 301 DYYERYTTTIFVRFIRLIFFVISLLLPGCFVAIILYHKEMIPTPLLISIMGAAHGVPFPIFIEALLMETAFEALREAGIRLPSPANQTVGIVGALVIGDAAVRAGVISPIMVIVIAITAIASFSIPSYDMGYAIRILRFSMLCLGAFLGL 450 451 YGVLLGIIVLLIHLSSLSSFGVNYLSPLAPLNLKELKDILIRFPWPHMKCRPHFANNNNLHR**k**KSSYENGKDEKG* 525 |
| **11655**  ***prfB*** | **E G** | 463/1107: A $\to$ T  1 atgataattgatttggaagaatccctatcaaaattgagcatattaaaaaaaacattaaataaaataaaggagtctctt***t***gacctaggtaactataagaataggatagaagaattgcagatgaaaatgcaggaacctaatttctgggatca 150 151 cttggaaagggcacaggaagtaacttctgaggaaaagtttttaaatgaaaagctggatatgtataataagttggagagtagaataaatgatgcagaaattctagctcatattgcggaagaagaggaagacgtttctgactgcaaagatat 300 301 attatctgaagtggaagatatcgaaaatataattgataaacttaaaatagaaatacttttatcaggagaatatgataaaaacaatgccatattgaatttacatgtgggagtaggtggcacagatgcccaagactggacggagatgctttt 450 451 gaggatgtatacc**T**gatgggcagaaaagtctgggtataaagttgagactttagatattcttcctgcagatgatgctggaataaaaagtgtatcattaaggataataggggaatttgcttatgggtatttgaaagccgaaaagggtattca 600 601 tagattggttagaatatcacctttcaatgctaatgggaaaaggcagacttcttttgcatctgtagaggttttgccggaacttaccaaagaccaagacatagatataaggccagaagatttaagggtagatacctataggtcttctggtgc 750 751 aggcggacagcacgtaaataaaacggaatctgcagtcaggataactcatatacctacgggtatagttgtacagtgccagaatgaaagaagtcagcattataatagagagactgcaatgctcatgttaaaatcaaaacttgtggaacttaa 900 901 agaaagagcacataaggaaaagatagaagatcttgcaggagaacttaaggacatgggatggggaagccagataagatcctatgtattccatccatatactcttgtaaaagatcatagaactggagtagagaatgggaatgtgtcttcagt 1050 1051 aatggatggagaaattgataattttatattagcctatttaagacagcaggcaaagtaa 1107  155/368: Arg $\to$ * (N/A)  1 MIIDLEESLSKLSILKKTLNKIKESLDLGNYKNRIEELQMKMQEPNFWDHLERAQEVTSEEKFLNEKLDMYNKLESRINDAEILAHIAEEEEDVSDCKDILSEVEDIENIIDKLKIEILLSGEYDKNNAILNLHVGVGGTDAQDWTEMLL 150 151 RMYT*****WAEKSGYKVETLDILPADDAGIKSVSLRIIGEFAYGYLKAEKGIHRLVRISPFNANGKRQTSFASVEVLPELTKDQDIDIRPEDLRVDTYRSSGAGGQHVNKTESAVRITHIPTGIVVQCQNERSQHYNRETAMLMLKSKLVELK 300 301 ERAHKEKIEDLAGELKDMGWGSQIRSYVFHPYTLVKDHRTGVENGNVSSVMDGEIDNFILAYLRQQAK* 368 |

**Table S3.** continued…

| **Gene** | **Ev.** | **Sequence Wild type:** dna **\|** AA **Variant: DNA \| aa** |
| --- | --- | --- |
| **11655**  ***prfB*** | **D** | 974/1107: G $\to$ T  1 atgataattgatttggaagaatccctatcaaaattgagcatattaaaaaaaacattaaataaaataaaggagtctctt***t***gacctaggtaactataagaataggatagaagaattgcagatgaaaatgcaggaacctaatttctgggatca 150 151 cttggaaagggcacaggaagtaacttctgaggaaaagtttttaaatgaaaagctggatatgtataataagttggagagtagaataaatgatgcagaaattctagctcatattgcggaagaagaggaagacgtttctgactgcaaagatat 300 301 attatctgaagtggaagatatcgaaaatataattgataaacttaaaatagaaatacttttatcaggagaatatgataaaaacaatgccatattgaatttacatgtgggagtaggtggcacagatgcccaagactggacggagatgctttt 450 451 gaggatgtataccagatgggcagaaaagtctgggtataaagttgagactttagatattcttcctgcagatgatgctggaataaaaagtgtatcattaaggataataggggaatttgcttatgggtatttgaaagccgaaaagggtattca 600 601 tagattggttagaatatcacctttcaatgctaatgggaaaaggcagacttcttttgcatctgtagaggttttgccggaacttaccaaagaccaagacatagatataaggccagaagatttaagggtagatacctataggtcttctggtgc 750 751 aggcggacagcacgtaaataaaacggaatctgcagtcaggataactcatatacctacgggtatagttgtacagtgccagaatgaaagaagtcagcattataatagagagactgcaatgctcatgttaaaatcaaaacttgtggaacttaa 900 901 agaaagagcacataaggaaaagatagaagatcttgcaggagaacttaaggacatgggatggggaagccagataa**T**atcctatgtattccatccatatactcttgtaaaagatcatagaactggagtagagaatgggaatgtgtcttcagt 1050 1051 aatggatggagaaattgataattttatattagcctatttaagacagcaggcaaagtaa 1107  325/368: Arg $\to$ Ile (0.139)  1 MIIDLEESLSKLSILKKTLNKIKESLDLGNYKNRIEELQMKMQEPNFWDHLERAQEVTSEEKFLNEKLDMYNKLESRINDAEILAHIAEEEEDVSDCKDILSEVEDIENIIDKLKIEILLSGEYDKNNAILNLHVGVGGTDAQDWTEMLL 150 151 RMYTRWAEKSGYKVETLDILPADDAGIKSVSLRIIGEFAYGYLKAEKGIHRLVRISPFNANGKRQTSFASVEVLPELTKDQDIDIRPEDLRVDTYRSSGAGGQHVNKTESAVRITHIPTGIVVQCQNERSQHYNRETAMLMLKSKLVELK 300 301 ERAHKEKIEDLAGELKDMGWGSQI**i**SYVFHPYTLVKDHRTGVENGNVSSVMDGEIDNFILAYLRQQAK* 368 |
| **13810**  ***thrB*** | **D** | 791/900: A $\to$ G  1 atgactaaagtcaaagttagggtgccggcgactacagcaaatatgggaccgggatttgatactttaggtatggcactcaaattatacaatgaaattgaagttgaagaaataactggaaaaactgaaatatataatggtggattaaagtta 150 151 gaagaggattttagggaaaatcttatataccaaagcatagtgagtgctatgaatgaacaaggatattcttataatggatttaaaataaatgtgcttaaatgtgatatacctatgtcaagagggcttggaagtagttctgcatgtatagta 300 301 ggtggtataacagctgcaaatgcaattatgaaaaataaaatggatatggaagatgtaattgatttggctacgaaaatagagggacatcctgataatgttgtacctgcagcacttggaggaatggtaatatctataaaagtgggcgaggat 450 451 ataaaacattcaaaggtaaatgtaccagataagttaaaatttgttgctatgataccatcttttaaggttagtacagctctttctagggaagttctacctaaatcttatttaaaagaggactgtatatttaacacttctaggtctgctatg 600 601 cttataagtgctctttataacaatgaatttgacaaacttagaatatgttttgaagataaaatccatcaaccttatagaaaaagtttaataagaaattttgatgatgtttttaaaaaatccaaagacttaggatctataggagaatttata 750 751 agtggttcaggatctacacttatggcagtagtggataaaa**G**tgcagagaaatttgtaagttccatgaaaaatttcttaagtgaactagaagatacttggaaggttattttacttgatccagatttacagggagctagagttttaaattag 900  264/299: Asn $\to$ Ser (0.355)  1 MTKVKVRVPATTANMGPGFDTLGMALKLYNEIEVEEITGKTEIYNGGLKLEEDFRENLIYQSIVSAMNEQGYSYNGFKINVLKCDIPMSRGLGSSSACIVGGITAANAIMKNKMDMEDVIDLATKIEGHPDNVVPAALGGMVISIKVGED 150 151 IKHSKVNVPDKLKFVAMIPSFKVSTALSREVLPKSYLKEDCIFNTSRSAMLISALYNNEFDKLRICFEDKIHQPYRKSLIRNFDDVFKKSKDLGSIGEFISGSGSTLMAVVDK**s**AEKFVSSMKNFLSELEDTWKVILLDPDLQGARVLN* 299 |
| **14845**  ***argR*** | **A G** | 271/456: AATTT $\to$ TATTC  1 atgaaaactgagagacatacaaaaatacttgaaattattaattcaaaggatatagaaacacaggaggagctagtagaagagcttaagagtgcgggaatagaagtaactcaagccactgtgtcaagagatataaagaaattaaaaattaca 150 151 aaagtagtgggccaaaatgggaaatcgaaatattctgttgtaaggcatacaggaaaactatttcctgataaaatagtagctatattttcacaaacaattattgatgtacaaactatgaaa**TATTC**tgttgtaataaaaactttatctggt 300 301 tcagccaaggcagcagcagaagctgcagactctttagctttcagcggggttattggcactgtagctggcaataatacattatttgtaataacaacagatgaaaaagcagccttgagtttggctaaaaaaattaaaaacatgatttctaat 450 451 caataa 456  91/151: AsnPhe $\to$ TyrSer (0.251 & 0.152)  1 MKTERHTKILEIINSKDIETQEELVEELKSAGIEVTQATVSRDIKKLKITKVVGQNGKSKYSVVRHTGKLFPDKIVAIFSQTIIDVQTMK**ys**VVIKTLSGSAKAAAEAADSLAFSGVIGTVAGNNTLFVITTDEKAALSLAKKIKNMISN 150 151 Q* 151 |
|  | **A^1^** | Base pair deletion calculated using per-base coverage  1 atgaaaactgagagacatacaaaaatacttgaaattattaattcaaaggatatagaaacacaggaggagctagtagaagagcttaagagtgcgggaatagaagtaactcaagccactgtgtcaagagat**ATAAAGAAATTAAAAATTACA** 150 151 **AAAGTAGTGGGCCAAAATGGGAAATCGAAATATTCTGTTGTAAGGCATACAGGAAAACTATTTCCTGATAAAATAGTAGCTATATTTTCACAAACAATTATTGATGTACAAACTATGAAAAATTTTGTTGTAATAAAAACTTTATCTGGT** 300 301 **TCAGCCAAGGC**agcagcagaagctgcagactctttagctttcagcggggttattggcactgtagctggcaataatacattatttgtaataacaacagatgaaaaagcagccttgagtttggctaaaaaaattaaaaacatgatttctaat 450 451 caataa  Translated peptide sequence of truncated protein when bases ≤ 25% normalised coverage are removed (from Expasy, open reading frames highlighted)  1 MKTERHTKILEIINSKDIETQEELVEELKSAGIEVTQATVSRDSSRSCRLFSFQRGYWHCSWQ-YIICNNNR-KSSLEFG-KN-KHDF-SI |
|  | **D^1^** | Base pair deletion calculated using per-base coverage  1 atgaaaactgagagacatacaaaaatacttgaaattattaattcaaaggatatagaaacacaggaggagctagtagaagagcttaagagtgcgggaatagaagtaactcaagccactgtgtcaagagatataaagaaattaaaaattaca 150 151 aaagtagtgggccaaaatgggaaa**TCGAAATATTCTGTTG**taaggcatacaggaaaactatttcctgataaaatagtagctatattttcacaaacaattattgatgtacaaactatgaaaaattttgttgtaataaaaactttatctggt 300 301 tcagccaaggcagcagcagaagctgcagactctttagctttcagcggggttattggcactgtagctggcaataatacattatttgtaataacaacagatgaaaaagcagccttgagtttggctaaaaaaattaaaaacatgatttctaat 450 451 caataa  Translated peptide sequence of truncated protein when bases ≤ 25% normalised coverage are removed (from Expasy, open reading frames highlighted)  1 MKTERHTKILEIINSKDIETQEELVEELKSAGIEVTQATVSRDIKKLKITKVVGQNGK-GIQENYFLIK--LYFHKQLLMYKL-KILL--KLYLVQPRQQQKLQTL-LSAGLLAL-LAIIHYL--QQMKKQP-VWLKKLKT-FLIN |

**Table S3.** continued…

| **Gene** | **Ev.** | **Sequence Wild type:** dna **\|** AA **Variant: DNA \| aa** |
| --- | --- | --- |
| **14845**  ***argR*** | **E^1^** | Base pair deletion calculated using per-base coverage  1 atgaaaactgagagacatacaaaaatacttgaaattattaattcaaaggatatagaaacacaggaggagctagtagaagagcttaagagtgcgggaatagaagtaactcaagccactgtgtcaagagatataaagaaattaaaaattaca 150 151 aaag**TAGTGGGCCAAAATGGGAAATCGAAATATTCTGTTGTAAGGCATACAGGAAAACTATTTCCTGATAAAATAGTAGCTATATTTTCACAAACAATTATTGATGTACAAACTATGAAAAATTTTGTTGTAATAAAAACTTTATCTGGT** 300 301 **TCAGCCAAGGCA**gcagcagaagctgcagactctttagctttcagcggggttattggcactgtagctggcaataatacattatttgtaataacaacagatgaaaaagcagccttgagtttggctaaaaaaattaaaaacatgatttctaat 450 451 caataa  Translated peptide sequence of truncated protein when bases ≤ 25% normalised coverage are removed (from Expasy, open reading frames highlighted)  1 MKTERHTKILEIINSKDIETQEELVEELKSAGIEVTQATVSRDIKKLKITKQGSSRSCRLFSFQRGYWHCSWQ-YIICNNNR-KSSLEFG-KN-KHDF-SI |
|  | **G^1^** | Base pair deletion calculated using per-base coverage  1 atgaaaactgagagacatacaaaaatacttgaaattattaattcaaaggatatagaaacacaggaggagctagtagaagagcttaagagtgcgggaatagaagtaactcaagccactgtgtcaagagatataaagaaattaaaaattaca 150 151 aaa**GTAGTGGGCCAAAATGGGAAATCGAAATATTCTGTTGTAAGGCATACAGGAAAACTATTTCCTGATAAAATAGTAGCTATATTTTCACAAACAATTATTGATGTACAAACTATGAAAAATTTTGTTGTAATAAAAACTTTATCTGGT** 300 301 **TCAGC**caaggcagcagcagaagctgcagactctttagctttcagcggggttattggcactgtagctggcaataatacattatttgtaataacaacagatgaaaaagcagccttgagtttggctaaaaaaattaaaaacatgatttctaat 450 451 caataa  Translated peptide sequence of truncated protein when bases ≤ 25% normalised coverage are removed (from Expasy, open reading frames highlighted)  1 MKTERHTKILEIINSKDIETQEELVEELKSAGIEVTQATVSRDIKKLKITKQGSSRSCRLFSFQRGYWHCSWQ-YIICNNNR-KSSLEFG-KN-KHDF-SI |

^1^Deletion of large fragment within argR was found using method described in **Figure S1**. Briefly, per-base coverage was normalized by the overall mean coverage of its alignment first, then Evolved lineages were normalized by WT. For these mutations bolded and capitalized bases represent bases where (normalized coverage of a lineage)/(normalized coverage of WT) are ≤ 25%, and underlined bases represent bases with no coverage (25% = 0.25 in **Figure S1**). Expasy [2] accessed via Swiss Institute of Bioinformatics’ Expasy bioinformatics resource portal.

Abbreviations: Ev. – evolved lineage, dna/DNA – deoxyribonucleic acid, aa/AA – amino acid. prfB has a programmed internal frameshift at the 79^th^ base pair (t), which is bolded and italicized. Exchangeability of amino acids ($EX$), found by Yampolsky and Stoltzfus [3], is shown in brackets. For reference to numerical values of $EX$, lysine is the most readily replaced amino acid ($\bar{EX}_{dest}$ = 0.409) and tryptophan is the least ($\bar{EX}_{dest}$ = 0.142), alanine is the best replacement ($\bar{EX}_{dest}$ =0.411) and tryptophan is the worst ($\bar{EX}_{dest}$ = 0.172), and the grand average of exchangeability is 0.29.

**Table S4.** CO₂/H₂ chemostat data at D = 0.5 day^-1^.

|  |  | **Evolved D** | | |  |  |  |  |  | **Control** |  |  |  |  |  |  |  |
| --- | --- | --- | --- | --- | --- | --- | --- | --- | --- | --- | --- | --- | --- | --- | --- | --- | --- |
|  |  | **BR1** | **±** | **BR2** | **±** | **BR3** | **±** | **Mean** | **±** | **BR1** | **±** | **BR2** | **±** | **BR3** | **±** | **Mean** | **±** |
| **Titer** (C-mM) | Biomass | 8.51 | 0.29 | 7.35 | 0.17 | 7.45 | 0.24 | **7.77** | **0.53** | 8.24 | 0.82 | 6.59 | 0.73 | 8.16 | 0.18 | **7.66** | **0.76** |
| mM | Acetate | 62.35 | 6.62 | 52.58 | 6.40 | 39.53 | 2.02 | **51.49** | **9.35** | 44.28 | 5.58 | 32.12 | 4.62 | 48.83 | 5.88 | **41.74** | **7.05** |
| mM | Ethanol | 45.90 | 2.07 | 41.39 | 4.15 | 45.30 | 1.65 | **44.20** | **2.00** | 48.92 | 7.19 | 46.20 | 6.90 | 58.66 | 6.56 | **51.26** | **5.35** |
| gDCW/L | Biomass | 0.204 | 0.007 | 0.176 | 0.004 | 0.179 | 0.006 | **0.186** | **0.013** | 0.198 | 0.020 | 0.158 | 0.018 | 0.196 | 0.004 | **0.184** | **0.018** |
| g/L | Acetate | 3.744 | 0.397 | 3.157 | 0.384 | 2.374 | 0.121 | **3.092** | **0.562** | 2.659 | 0.335 | 1.929 | 0.278 | 2.932 | 0.353 | **2.507** | **0.424** |
| g/L | Ethanol | 2.115 | 0.095 | 1.907 | 0.191 | 2.087 | 0.076 | **2.036** | **0.092** | 2.254 | 0.331 | 2.129 | 0.318 | 2.702 | 0.302 | **2.362** | **0.246** |
| **Off gas comp.** | H₂ | 0.664 | 0.001 | 0.667 | 0.002 | 0.657 | 0.001 | **0.662** | **0.004** | 0.667 | 0.002 | 0.670 | 0.002 | 0.669 | 0.001 | **0.668** | **0.001** |
| mol% | CO₂ | 0.213 | 0.001 | 0.211 | 0.002 | 0.199 | 0.001 | **0.208** | **0.006** | 0.221 | 0.002 | 0.220 | 0.002 | 0.217 | 0.001 | **0.219** | **0.001** |
|  | Ar | 0.123 | 0.000 | 0.122 | 0.001 | 0.145 | 0.002 | **0.130** | **0.010** | 0.113 | 0.003 | 0.110 | 0.003 | 0.114 | 0.001 | **0.112** | **0.002** |
| **Uptake rate** | H₂ | 271.58 | 5.17 | 262.53 | 4.12 | 280.96 | 8.04 | **271.69** | **7.52** | 256.39 | 42.46 | 211.32 | 46.04 | 265.28 | 14.30 | **244.33** | **23.62** |
| mM/day | CO₂ | 107.09 | 1.21 | 108.72 | 4.30 | 113.41 | 2.97 | **109.74** | **2.68** | 96.09 | 16.11 | 84.85 | 17.75 | 108.01 | 5.29 | **96.32** | **9.46** |
|  | Ethanol | -0.91 | 0.03 | -0.80 | 0.06 | -0.57 | 0.08 | **-0.76** | **0.14** | -1.25 | 0.12 | -1.23 | 0.05 | -1.26 | 0.11 | **-1.25** | **0.01** |
| **Ratio** | CO₂/H₂ | 0.39 | 0.01 | 0.41 | 0.02 | 0.40 | 0.02 | **0.40** | **0.01** | 0.37 | 0.09 | 0.40 | 0.12 | 0.41 | 0.03 | **0.39** | **0.01** |
| **q(gas)** | H₂ | 1329.11 | 52.02 | 1488.01 | 42.14 | 1572.06 | 68.33 | **1463.06** | **100.74** | 1296.30 | 250.58 | 1337.04 | 326.83 | 1354.60 | 79.12 | **1329.31** | **24.42** |
| mmol/gDCW/day | CO₂ | 524.09 | 18.87 | 616.24 | 28.35 | 634.59 | 26.60 | **591.64** | **48.35** | 485.85 | 94.76 | 536.83 | 127.11 | 551.52 | 29.70 | **524.74** | **28.14** |
|  | Ethanol | -4.45 | 0.20 | -4.53 | 0.34 | -3.21 | 0.45 | **-4.06** | **0.60** | -6.34 | 0.87 | -7.79 | 0.91 | -6.42 | 0.60 | **-6.85** | **0.67** |
| **Ratio** | CO₂/H₂ | 0.39 | 0.02 | 0.41 | 0.02 | 0.40 | 0.02 | **0.40** | **0.01** | 0.37 | 0.10 | 0.40 | 0.14 | 0.41 | 0.03 | **0.39** | **0.01** |
| **q(liq)** (C-mM/day) | Acetate | 62.35 | 6.62 | 52.58 | 6.40 | 39.53 | 2.02 | **51.5** | **9.4** | 44.28 | 5.58 | 32.12 | 4.62 | 48.83 | 5.88 | **41.7** | **7.1** |
| C-mM/day | Ethanol | 47.7 | 2.1 | 43.0 | 4.2 | 46.4 | 1.7 | **45.7** | **2.0** | 51.4 | 7.2 | 48.7 | 6.9 | 61.2 | 6.6 | **53.8** | **5.4** |
| C-mM/day | Biomass | 4.3 | 0.1 | 3.7 | 0.1 | 3.7 | 0.1 | **3.9** | **0.3** | 4.1 | 0.4 | 3.3 | 0.4 | 4.1 | 0.1 | **3.8** | **0.4** |
| mmol/gDCW/day | Acetate | 152.6 | 17.0 | 149.0 | 18.5 | 110.6 | 6.7 | **137.4** | **19.0** | 111.9 | 18.0 | 101.6 | 18.5 | 124.7 | 15.3 | **112.7** | **9.4** |
| mmol/gDCW/day | Ethanol | 116.8 | 8.5 | 121.8 | 15.5 | 129.9 | 19.1 | **122.8** | **5.4** | 130.0 | 29.2 | 154.0 | 33.9 | 156.2 | 23.0 | **146.7** | **11.8** |

**Tables S4.** continued…

|  |  | **Evolved D** | | |  |  |  |  |  | **DSM19630** | | |  |  |  |  |  |
| --- | --- | --- | --- | --- | --- | --- | --- | --- | --- | --- | --- | --- | --- | --- | --- | --- | --- |
|  |  | **BR1** | **±** | **BR2** | **±** | **BR3** | **±** | **Mean** | **±** | **BR1** | **±** | **BR2** | **±** | **BR3** | **±** | **Mean** | **±** |
| **C-balance** | CO₂_,liq_ | 2.92 | 0.01 | 2.89 | 0.03 | 2.72 | 0.02 | **2.84** | **0.09** | 3.02 | 0.02 | 3.02 | 0.02 | 2.98 | 0.01 | **3.01** | **0.02** |
|  | Cysteine | 3.52 |  | 3.52 |  | 3.52 |  | **3.52** | **0.00** | 3.52 |  | 3.52 |  | 3.52 |  | **3.52** | **0.00** |
|  | **In** | 107.69 | 1.21 | 109.35 | 4.30 | 114.21 | 2.97 | **110.42** | **2.77** | 96.59 | 16.11 | 85.35 | 17.75 | 108.55 | 5.29 | **96.83** | **9.47** |
|  | **Out** | 113.42 | 6.93 | 98.44 | 7.63 | 89.12 | 2.61 | **100.33** | **10.01** | 98.58 | 9.11 | 82.85 | 8.31 | 112.82 | 8.81 | **98.08** | **12.24** |
|  | **Overall** | **105%** |  | **90%** |  | **78%** |  | **91%** | **9%** | **102%** |  | **97%** |  | **104%** |  | **101%** | **16%** |
|  | Biomass | 3.8% |  | 3.7% |  | 4.2% |  | 3.9% | 0.2% | 4.2% |  | 4.0% |  | 3.6% |  | 3.9% | 0.2% |
|  | Acetate | 55.0% |  | 53.4% |  | 44.4% |  | 50.9% | 4.7% | 44.9% |  | 38.8% |  | 43.3% |  | 42.3% | 2.6% |
|  | Ethanol | 40.5% |  | 42.0% |  | 50.8% |  | 44.4% | 4.6% | 49.6% |  | 55.8% |  | 52.0% |  | 52.5% | 2.5% |
| **Growth rate** |  | 0.514 | 0.030 | 0.495 | 0.045 | 0.511 | 0.069 | **0.51** | **0.01** | 0.48 | 0.08 | 0.47 | 0.05 | 0.51 | 0.04 | **0.49** | **0.02** |

**Table S5.** CO₂/H₂ chemostat data near Dₘₐₓ.

|  |  | **Evolved D (D = 0.90 day^-1^)** | | | | | | **DSM19630 (same BR, BR1 0.60 and BR2 0.65 day^-1^)** | | | | |  |
| --- | --- | --- | --- | --- | --- | --- | --- | --- | --- | --- | --- | --- | --- |
|  |  | **BR1** | **±** | **BR2** | **±** | **Mean** | **±** | **BR1** | **±** | **BR2** | **±** | **Mean** | **±** |
| **Titer** (C-mM) | Biomass | 8.42 | 0.33 | 8.68 | 0.11 | **8.55** | **0.13** | 9.16 | 0.08 | 7.85 | 0.04 |  |  |
| mM | Acetate | 128.86 | 1.39 | 129.56 | 2.06 | **129.21** | **0.35** | 63.82 | 1.96 | 110.77 | 0 |  |  |
| mM | Ethanol | 0.00 | 0.00 | 0.00 | 0.00 | **0.00** | **0.00** | 50.87 | 1.11 | 1.58 | 0 |  |  |
| gDCW/L | Biomass | 0.202 | 0.008 | 0.208 | 0.003 | **0.21** | **0.00** | 0.220 | 0.002 | 0.188 | 0.001 |  |  |
| g/L | Acetate | 7.738 | 0.084 | 7.780 | 0.124 | **7.76** | **0.02** | 3.833 | 0.117 | 6.652 | 0.000 |  |  |
| g/L | Ethanol | 0.000 | 0.000 | 0.000 | 0.000 | **0.00** | **0.00** | 2.344 | 0.051 | 0.073 | 0.000 |  |  |
| **Uptake rate** | H₂ | 443.38 | 17.91 | 534.54 | 21.33 | **488.96** | **45.58** | 366.84 | 4.12 | 314.88 | 3.24 | **340.86** | **25.98** |
| mM/day | CO₂ | 204.87 | 1.98 | 259.85 | 3.79 | **232.36** | **27.49** | 159.76 | 1.18 | 154.56 | 1.23 | **157.16** | **2.60** |
|  | Ethanol | -0.019 | 0.004 | -0.012 | 0.004 | **-0.02** | **0.00** | -1.34 | 0.05 | -0.02 | 0.00 |  |  |
| **Ratio** | CO₂/H₂ | 0.462 | 0.02 | 0.486 | 0.02 | **0.47** | **0.01** | 0.44 | 0.01 | 0.49 | 0.01 | **0.46** | **0.03** |
| **q(gas)** | H₂ | 2193.28 | 123.90 | 2567.23 | 107.71 | **2380.26** | **186.97** | 1667.85 | 23.48 | 1671.61 | 19.19 | **1669.73** | **1.88** |
| mmol/gDCW/day | CO₂ | 1013.45 | 41.20 | 1248.00 | 24.35 | **1130.72** | **117.28** | 726.35 | 8.15 | 820.49 | 7.76 | **773.42** | **47.07** |
|  | Ethanol | -0.09 | -0.02 | -0.06 | -0.02 | **-0.08** | **0.02** | -6.09 | -0.21 | -0.11 | 0.00 |  |  |
| **Ratio** | CO₂/H₂ | 0.462 | 0.03 | 0.486 | 0.02 | **0.47** | **0.01** | 0.436 | 0.01 | 0.491 | 0.01 | **0.46** | **0.03** |
| **q(liq)** (C-mM/day) | Acetate | 231.95 | 2.51 | 233.20 | 3.71 | **232.57** | **0.63** | 76.58 | 2.35 | 144.00 | 0.00 |  |  |
| C-mM/day | Ethanol | 0.02 | 0.00 | 0.01 | 0.00 | **0.02** | **0.00** | 63.72 | 1.42 | 2.10 | 0.00 |  |  |
| C-mM/day | Biomass | 7.58 | 0.30 | 7.81 | 0.10 | **7.69** | **0.11** | 5.50 | 0.05 | 5.10 | 0.03 |  |  |
| mmol/gDCW/day | Acetate | 573.69 | 23.5 | 560.00 | 11.5 | **566.84** | **6.84** | 174.10 | 5.5 | 382.23 | 2.0 |  |  |
| mmol/gDCW/day | Ethanol | 0.09 | 0.0 | 0.06 | 0.0 | **0.08** | **0.02** | 144.86 | 3.0 | 5.47 | 0.0 |  |  |

**Table S5.** continued…

|  |  | **Evolved D (D = 0.90 day^-1^)** | | |  |  |  | **DSM19630 (same BR, BR1 0.60 and BR2 0.65 day^-1^)** | | | | | |
| --- | --- | --- | --- | --- | --- | --- | --- | --- | --- | --- | --- | --- | --- |
|  |  | **BR1** | **±** | **BR2** | **±** | **Mean** | **±** | **BR1** | **±** | **BR2** | **±** | **Mean** | **±** |
| **C-bal.** C-mM/day | CO_2,liq_ | 3.01 | 0.14 | 3.01 | 0.13 | **3.01** | **0.00** | 3.01 | 0.04 | 3.01 | 0.03 |  |  |
| C-mM/day | Cysteine | 3.52 |  | 3.52 |  | **3.52** |  | 3.52 |  | 3.52 |  |  |  |
| C-mM/day | **In** | 205.38 | 41.20 | 260.36 | 24.35 | **232.87** | **27.49** | 160.26 | 8.15 | 155.06 | 7.76 |  |  |
| C-mM/day | **Out** | 239.55 | 1.40 | 241.02 | 2.06 | **240.28** | **0.74** | 145.81 | 2.26 | 151.20 | 0.02 |  |  |
| **%** | **Overall** | **117%** |  | **93%** |  | **103%** | **12%** | **91%** | **5%** | **98%** | **5%** |  |  |
| **%_norm_** | Biomass | 3.2% |  | 3.2% |  | **3.2%** | **0.0%** | 3.8% |  | 3.4% |  |  |  |
| **%_norm_** | Acetate | 96.8% |  | 96.8% |  | **96.8%** | **0.0%** | 52.5% |  | 95.2% |  |  |  |
| **%_norm_** | Ethanol | 0.0% |  | 0.0% |  | **0.0%** | **0.0%** | 43.7% |  | 2.8% |  |  |  |
| **µ day^-1^** |  | 0.919 | 0.042 | 0.926 | 0.039 | **0.922** | **0.004** | 0.592 | 0.010 | 0.646 | 0.004 |  |  |

**Table S6.** DE proteins between WT CO/CO₂/H₂ and CO₂/H₂ fermentations.

| **Locus** | **1** | **2** | **3** | **Protein name** | **KO Description** | **Description** | **#U.P.** | $\boldsymbol{p}$**-value** | **CO/**  **CO₂** | **Ev./**  **WT** |
| --- | --- | --- | --- | --- | --- | --- | --- | --- | --- | --- |
| 13810 |  |  | Glycine, serine and threonine metabolism | thrB | homoserine kinase | Homoserine kinase | 10 | 3.5E-03 | -1.43 | -2.74 |
| 11785 |  |  | Phenylalanine metabolism | mhpD | 2-oxopent-4-enoate hydratase | 2-oxopent-4-enoate hydratase | 12 | 8.0E-03 | 2.57 |  |
| 00595 |  |  | Valine, leucine and isoleucine biosynthesis | ilvH | acetolactate synthase small subunit | acetolactate synthase, small subunit | 8 | 3.4E-04 | -1.76 | -0.90 |
| 14770 |  |  | Wood-Ljungdahl pathway | cooS1a | 4Fe-4S ferredoxin, iron-sulfur-binding | 4Fe-4S ferredoxin, iron-sulpur binding domain-c | 17 | 2.7E-03 | -1.47 |  |
| 10985 |  |  | Amino sugar and nucleotide sugar metabolism | abfA | alpha-N-arabinofuranosidase | alpha-L-arabinofuranosidase domain protein | 2 | 2.1E-02 | -1.13 | -2.32 |
| 10710 |  |  | Glyoxylate and dicarboxylate metabolism | garR | 2-hydroxy-3-oxopropionate reductase | 2-hydroxy-3-oxopropionate reductase | 6 | 1.8E-03 | -1.53 | -1.74 |
| 10970 |  |  | Pentose and glucuronate interconversions | araD | L-ribulose-5-phosphate 4-epimerase AraD | L-ribulose-5-phosphate 4-epimerase | 3 | 6.7E-03 | -1.33 |  |
| 00365 |  |  | Bacterial chemotaxis | mcp8 | methyl-accepting chemotaxis protein | methyl-accepting chemotaxis sensory transducer | 40 | 2.1E-06 | -2.37 | -3.67 |
| 01675 |  |  | Bacterial chemotaxis | mcp4 | methyl-accepting chemotaxis protein | methyl-accepting chemotaxis sensory transducer | 3 |  |  |  |
| 11095 |  |  | Bacterial chemotaxis | motB2 | chemotaxis protein MotB | Motility protein B, N-terminal domain containing | 2 |  |  |  |
| 15000 |  |  | Flagellar assembly | fliS | flagellar export chaperone FliS | flagellar protein FliS | 4 | 1.4E-03 | 2.83 |  |
| 00490 |  |  | Acetate & ethanol production | AOR2 | aldehyde ferredoxin oxidoreductase | Aldehyde ferredoxin oxidoreductase | 52 | 3.8E-06 | -2.31 |  |
| 02630 |  |  | Acetate & ethanol production | adh3 | NADPH-dependent butanol dehydrogenase | Alcohol dehydrogenase | 12 | 1.2E-12 | -3.64 | -1.70 |
| 07665 |  |  | Energy conservation | Nfn | Nfn transhydrogenase | glutamate synthase (NADPH), homotetrameric | 73 | 1.3E-02 | -1.21 |  |
| 07645 |  |  | Hydrogenases | Hyd1a | hydrogenase Fe-only | hydrogenase, Fe-only | 31 | 3.2E-02 | -1.05 | -2.09 |
| 07650 |  |  | Hydrogenases | Hyd1b | NADH-quinone oxidoreductase subunit NuoF | NADH dehydrogenase (quinone) | 40 | 5.8E-03 | -1.35 | -2.43 |
| 07655 |  |  | Hydrogenases | Hyd1c | NAD(P)H-dependent oxidoreductase subunit E | NADH dehydrogenase (ubiquinone) 24 kDa subunit | 7 | 2.7E-03 | -1.47 | -2.89 |
| 00495 |  |  | Sulphur relay system | moaD2 | thiamine S protein | thiamine S protein | 2 | 2.5E-03 | -1.48 |  |
| 00500 |  |  | Sulphur relay system | moeB2 | molybdopterin biosynthesis protein MoeB | UBA/THIF-type NAD/FAD binding protein | 3 | 1.3E-04 | -1.89 |  |
| 13380 |  |  | ABC transporters | metQ2 | ABC transporter periplasmic protein | NLPA lipoprotein | 2 | 2.0E-04 | -1.83 |  |
| 11330 |  |  | ABC transporters | lacE | carbohydrate ABC transporter substrate-binding | extracellular solute-binding protein family 1 | 3 |  |  |  |
| 11775 |  |  | Nicotinate and nicotinamide metabolism | nadC2 | Quinolinate phosphoribosyl transferase | Quinolinate phosphoribosyl transferase | 2 | 1.4E-03 | 2.83 |  |
| 00120 |  |  | No KO ID | RS00120 | flavin reductase-like protein | Rubredoxin domain containing protein | 22 | 2.0E-16 | -4.19 | -3.73 |
| 07615 |  |  | No KO ID | RS07615 | methyl-accepting chemotaxis sensory transducer | methyl-accepting chemotaxis sensory transducer | 8 | 1.8E-04 | -1.85 | -1.91 |
| 07785 |  |  | No KO ID | RS07785 | RNA-binding protein | RNA-binding protein | 2 | 6.4E-05 | 3.24 | 1.81 |
| 08610 |  |  | No KO ID | RS08610 | NADH peroxidase | Rubrerythrin | 32 | 9.4E-08 | -2.69 | -1.27 |
| 19150 |  |  | No KO ID | RS19150 | molybdopterin-binding protein | molybdopterin binding domain-containing protein | 23 | 1.4E-08 | 4.13 | 0.75 |
| 07625 |  |  | Purine metabolism | IMPDH | IMP dehydrogenase | IMP dehydrogenase | 45 | 2.9E-02 | -1.07 | -0.73 |
| 07395 |  |  | Function unknown | K06926 | hypothetical protein | hypothetical protein | 5 |  |  |  |
| 00950 |  |  | Transporters | ydjE2 | MFS transporter | General substrate transporter | 6 |  |  |  |
| 11770 |  |  | Transporters | benE | benzoate transporter | Benzoate membrane transport protein | 2 | 2.2E-03 | 2.77 |  |
| 09580 |  |  | DNA repair and recombination proteins | radA | DNA repair protein RadA | DNA repair protein RadA | 2 |  |  |  |
| 14330 |  |  | DNA replication | dnaG | DNA primase | DNA primase | 2 |  |  |  |
| 12765 |  |  | Two-component system | E3.1.3.48 | protein-tyrosine-phosphatase | Protein-tyrosine-phosphatase | 2 | 2.9E-04 | 3.05 |  |
| 02250 |  |  | Transcription factors | acoR7 | sigma54 specific transcriptional regulator wit | sigma54 specific transcriptional regulator with | 2 |  |  |  |

**Table S6.** continued…

| **Locus** | **1** | **2** | **3** | **Protein name** | **KO Description** | **Description** | **#U.P.** | $\boldsymbol{p}$**-value** | **CO/**  **CO₂** | **Ev./**  **WT** |
| --- | --- | --- | --- | --- | --- | --- | --- | --- | --- | --- |
| 02615 |  |  | Transcription factors | acoR8 | sigma-54-dependent Fis family transcriptional | PAS modulated sigma54 specific transcriptional | 2 |  |  |  |
| 09325 |  |  | Ribosome | rpmJ | 50S ribosomal protein L36 | 50S ribosomal protein L36 | 2 | 3.5E-06 | -2.31 |  |
| 09375 |  |  | Ribosome | rpsN | type Z 30S ribosomal protein S14 | Ribosomal protein S14, type Z | 3 | 1.8E-06 | -2.39 |  |
| 09515 |  |  | Ribosome | rpmG | 50S ribosomal protein L33 | 50S ribosomal protein L33 | 3 | 8.1E-05 | -1.95 | 0.71 |
| 14600 |  |  | Ribosome biogenesis | rumA2 | 23S rRNA (uracil(1939)-C(5))-methyltransferase | RNA methyltransferase, TrmA family | 3 | 2.8E-02 | -1.08 | -0.94 |
| 08820 |  |  | Amino acid metabolism | eutQ | ethanolamine utilization protein EutQ | Ethanolamine utilization EutQ family protein | 2 | 1.1E-02 | 2.52 | 1.13 |
| 04220 |  |  | Energy metabolism | fprB | hypothetical protein | Rubrerythrin | 19 | 5.6E-04 | -1.7 | -1.18 |
| 00125 |  |  | Enzymes with EC numbers | ftnA | ferritin | Ferroxidase | 15 | 2.0E-16 | -4.14 | -3.76 |
| 00370 |  |  | Enzymes with EC numbers | draG | ADP-ribosyl-[dinitrogen reductase] hydrolase | ADP-ribosyl-(dinitrogen reductase) hydrolase | 4 | 8.8E-03 | -1.28 | -2.32 |
| 01375 |  |  | Enzymes with EC numbers | fprA1_2 | FprA family A-type flavoprotein | beta-lactamase domain protein | 56 | 1.8E-12 | -3.6 | -4.42 |
| 08860 |  |  | Enzymes with EC numbers | cutC | choline trimethylamine-lyase | pyruvate formate-lyase PFL | 26 | 7.3E-03 | 2.58 | 0.81 |
| 07290 |  |  | Others | hr2 | hemerythrin | hemerythrin-like metal-binding protein | 18 | 2.0E-16 | -4.56 | -4.00 |
| 07715 |  |  | Signalling proteins | cstA | carbon starvation protein A | carbon starvation protein CstA | 5 | 2.0E-05 | -2.12 | -2.05 |
| 00375 |  |  |  |  |  | 3-oxoacyl-(acyl-carrier-protein) reductase | 9 | 2.3E-09 | -3.03 | -4.39 |
| 00975 |  |  |  |  |  | 2,4-dienoyl-CoA reductase (NADPH) | 5 | 8.4E-07 | -2.47 | 0.96 |
| 03920 |  |  |  |  |  | diguanylate cyclase | 2 |  |  |  |
| 04215 |  |  |  |  |  | hypothetical protein | 15 | 2.1E-08 | -2.83 |  |
| 06565 |  |  |  |  |  | Protein of unknown function DUF4364 | 2 |  |  |  |
| 09935 |  |  |  |  |  | dUTPase | 2 |  |  |  |
| 10705 |  |  |  |  |  | type III effector Hrp-dependent outer protein | 3 | 8.0E-04 | -1.65 | -2.24 |
| 11890 |  |  |  |  |  | hypothetical protein | 2 | 1.3E-02 | -1.21 | 1.28 |
| 13995 |  |  |  |  |  | ferredoxin | 6 | 4.6E-05 | -2.02 |  |
| 08865 |  |  |  |  |  | Acetaldehyde dehydrogenase (acetylating) | 3 |  |  |  |


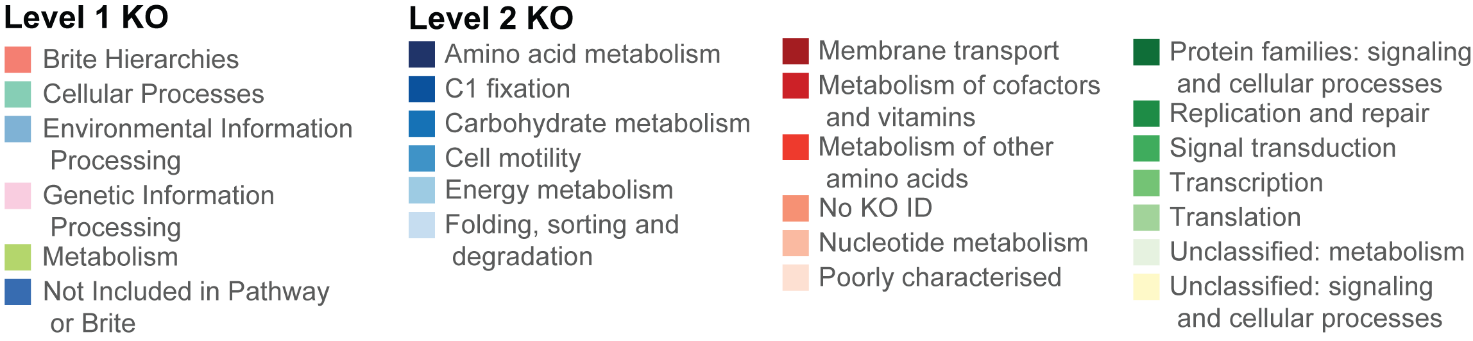


Locus numbers are preceded by “CAETHG_RS”; “1”,”2”, and “3” refer to Kegg Orthology Level 1, 2, and 3 from Valgepea et al. [22], where Level 1 and 2 match legend below table; “Protein name” and “KO Description” are also from Valgepea et al. [1], “#U.P.” is number of unique peptides; fold changes are in ${log}_{2} FC$; “CO/CO₂” shows fold change between WT CO/CO₂/H₂ and CO₂/H₂ fermentations; “Ev./WT” shows matching fold change if protein is also a DE protein between Evolved D and WT CO₂/H₂ fermentations; grey-filled locus numbers indicate genes that are on/off expressed and therefore have no FC or $p$-value associated (but FC colour indicates in what condition gene is expressed – green is CO/CO₂/H₂); scale colors are between -6.64-red, 0-yellow, and 6.64-green.

**References**

[1] K. Valgepea, G. Talbo, N. Takemori, A. Takemori, C. Ludwig, V. Mahamkali, A.P. Mueller, R. Tappel, M. Köpke, S.D. Simpson, L.K. Nielsen, E. Marcellin, Absolute Proteome Quantification in the Gas-Fermenting Acetogen *Clostridium autoethanogenum*, MSystems. 7 (2022). https://doi.org/10.1128/msystems.00026-22.

[2] E. Gasteiger, A. Gattiker, C. Hoogland, I. Ivanyi, R.D. Appel, A. Bairoch, ExPASy: The proteomics server for in-depth protein knowledge and analysis, Nucleic Acids Res. 31 (2003) 3784–3788. https://doi.org/10.1093/nar/gkg563.

[3] L.Y. Yampolsky, A. Stoltzfus, The exchangeability of amino acids in proteins, Genetics. 170 (2005) 1459–1472. https://doi.org/10.1534/genetics.104.039107.
