# Supplementary material for: Adaptive laboratory evolution of Clostridium autoethanogenum to metabolize CO2 and H2 enhances growth rates in chemostat and unravels proteome and metabolome alterations
Source: Microb Biotechnol. 2024 Apr 3;17(4):e14452. doi: 10.1111/1751-7915.14452 (PMC10990044; doi:10.1111/1751-7915.14452)
Supplement: Supplementary file 3 — Appendices S1–S3. [file MBT2-17-e14452-s003.zip › mbt214452-sup-0004-AppendixS1-S2.docx]

Supporting file for

Lab-evolution of *Clostridium autoethanogenum* modifies proteome of CO₂/H₂ chemostat fermentation while metabolites control phenotype.

James Heffernan^a,b^, Ricardo A. Garcia Gonzalez^a,b^, Vishnu Mahamkali^c^, Tim McCubbin^d^, Dara Daygon^d^, Lian Liu^d^, Robin Palfreyman^d^ Audrey Harris^c^, Michael Koepke^c^, Kaspar Valgepea^f^, Lars Nielsen^a,b,d,e^, Esteban Marcellin^a,b,d,*^

^a^ Australian Institute of Bioengineering and Nanotechnology, The University of Queensland, Corner Cooper Rd & College Rd, St. Lucia, QLD 4072, Australia. ^b^ ARC Centre of Excellence in Synthetic Biology, The University of Queensland, St. Lucia, QLD 4072, Australia. ^c^ LanzaTech Inc., Skokie IL 60077, United States. ^d^ Queensland Metabolomics and Proteomics Q-MAP, The University of Queensland, St. Lucia QLD 4072, Australia. ^e^ The Novo Nordisk Foundation Center for Biosustainability, Technical University of Denmark, DK-2800 Kgs. Lyngby, Denmark. ^f^ ERA Chair in Gas Fermentation Technologies, Institute of Technology, University of Tartu, 50411 Tartu, Estonia.

Contents

[1. Appendix S1: Supporting Experimental Procedures 2](#_Toc155195191)

[1.1. Whole population genome sequencing and analysis 2](#_Toc155195192)

[DNA extraction 2](#_Toc155195193)

[Library Preparation 2](#_Toc155195194)

[Library Pooling, QC and Loading 2](#_Toc155195195)

[1.2. Proteomic analysis 2](#_Toc155195196)

[Data Dependent Acquisition (DDA) protein extraction 2](#_Toc155195197)

[Data Independent Acquisition (DIA) protein extraction 3](#_Toc155195198)

[LC method for DDA and DIA mass spectrometry 3](#_Toc155195199)

[DDA mass spectrometry 3](#_Toc155195200)

[DIA mass spectrometry 3](#_Toc155195201)

[DDA mass spectrometry data analysis 4](#_Toc155195202)

[DIA mass spectrometry data analysis 4](#_Toc155195203)

[2. Appendix S2: Supporting gene variant analysis through alignment functions. 5](#_Toc155195204)

# Appendix S1: Supporting Experimental Procedures

## Whole population genome sequencing and analysis

### DNA extraction

DNA was extracted from 50-200 mg of sample with a preliminary step of bead beating using 0.1 dia. glass beads (BioSpec Products #11079101) on the Powerlyser 24 homogenizer (Mo-Bio #13155). The sample was added to a bead tube filled with 850 µL of CD1 (Qiagen cat #47016) and vortexed to mix. The tubes were heated at 65 °C for 10 minutes. The sample was then bead beat for five minutes at 2,000 RPM then centrifuged for one minute at 15,000 g. The resulting lysate was transferred to a new collection tube. Extraction was as per Qiagen DNeasy Powersoil Pro Kit (cat #47016). The final elution volume was 50 µl, with concentrations (quantified with a Qubit) of 7.2, 8.7, 3.1, 7.9 and 3.93 ng/µL for parental, Evolved A, D, E and G. Samples were normalized to a total input of 6 ng (at 1 ng/µL) for the library preparation.

### Library Preparation

Libraries were prepared according to the manufacturer’s protocol using Nextera DNA Flex Library Preparation Kit (Illumina # 20018705) at Australian Centre for Ecogenomics. The only alterations to the protocol as outlined was the reduction of total reaction volume for processing in 96 well plate format. Library preparation was run on the Mantis Liquid Handler (Formulatrix). This covers “Tagment Genomic DNA" to "Amplify DNA” in the protocol (Mantis- Nextera DNA Flex library prep protocol). Resulting amplified libraries were cleaned-up as per the “Clean Up Libraries” section in the manufacturer’s protocol. On completion of the library prep protocol, each library was quantified, and QC was performed using the Quant-iT™ dsDNA HS Assay Kit (Invitrogen) and Agilent D1000 HS tapes (#5067-5582) on the TapeStation 4200 (Agilent # G2991AA) as per the manufacturer’s protocol.

### Library Pooling, QC and Loading

Nextera DNA Flex libraries were pooled at equimolar amounts of 0.5 nM per library to create a sequencing pool. The library pool was quantified in triplicates using the Qubit™ dsDNA HS Assay Kit (Invitrogen). Library QC is performed using the Agilent D1000 HS tapes (#5067-5582) on the TapeStation 4200 (Agilent # G2991AA) as per the manufacturer’s protocol. According to the manufacturer's protocol, the library was sequenced on a NovaSeq6000 (Illumina) on an v1.5 S4 flowcell and 2 x 150bp paired end chemistry.

## Proteomic analysis

### Data Dependent Acquisition (DDA) protein extraction

The collected samples were suspended in 500 µL 5% sodium dodecyl sulfate (SDS) lysis buffer (with DTT and Trizma base). Cell lysis was then performed by using glass beads and repeating a “lysis cycle” consisting of heating, bead beating, centrifugation, and vortexing before protein quantification with a colorific assay using Bradford reagent (Thermo Fischer). Following, the alkylation of cysteines was conducted by addition 80 µL 500 mM of iodoacetamide (IAA) and incubated in the dark for 30 min. The proteins were acidified with 25 µL a final concentration of 12% phosphoric acid and diluted with 3.3 mL S-Trap binding buffer to form colloidal protein particulate. Then the mix was transferred into S-Trap Mini Spin columns (Protifi), trapped in filter by centrifugations at 4,000 g for 1 min, then washed three times with 3 mL S-Trap binding buffer. The peptides were generated from a digestion process by adding digestion buffer containing 20 µg trypsin and 350 µL of 50 mM ammonium bicarbonate pH 8 on top of the S- traps. Then the S-trap was incubated in oven overnight at 37 °C. The digested peptides were eluted with 500 µL of 5% ACN in 0.1% formic acid, followed by 50% ACN in 0.1% formic acid and 75% ACN in 0.1% formic acid. All the elutions were dried and resuspended in 5% acetonitrile (ACN), 0.1 % formic acid (FA) prior to liquid chromatography.

### Data Independent Acquisition (DIA) protein extraction

The collected samples were solubilized in 50 µL 5% sodium dodecyl sulfate (SDS) lysis buffer. Then the proteins were reduced by adding 4 µL aliquot of 500 mM of dithiothreitol (DTT) and heating for 60 min at 70 °C. Following, the alkylation of cysteines was conducted by addition 8 µL 500 mM of iodoacetamide (IAA) and incubated in the dark for 30 min. The proteins were acidified with 2.5 µL a final concentration of 12% phosphoric acid and diluted with 165 µL S-Trap binding buffer to form colloidal protein particulate. Then the mix was transferred into S-Trap Micro Spin columns (Protifi), trapped in filter by centrifugations at 4,000 g for 1 min, then washed three times with 150 µL S-Trap binding buffer. The peptides were generated from a digestion process by adding digestion buffer containing 2 µg trypsin and 25 µL of 50 mM ammonium bicarbonate pH 8 on top of the S- traps. Then the S-trap was incubated in oven overnight at 37 °C. The digested peptides were eluted with 40 µL of 5% ACN in 0.1% formic acid, followed by 50% ACN in 0.1% formic acid and 75% ACN in 0.1% formic acid. All the elutions were dried and resuspended in 5% acetonitrile (ACN), 0.1 % formic acid (FA) prior to liquid chromatography.

### LC method for DDA and DIA mass spectrometry

The peptides were analyzed by liquid chromatography-mass spectrometry (LCMS). The HPLC system used was an Ultimate 3000 RSLCnano (ThermoFisher), where the mobile phase was H₂O (A) and 80% ACN (B), both in 0.1% formic acid. Initially, 2 µL of each sample was injected onto a ThermoFisher Acclaim PepMap C18 trap reversed-phase column (300 µm x 5 mm nano viper, 5 µm particle size) used for online desalting and washed at 20 µl/min, prior to a Waters nanoEaseTM M/Z CSH C18 resolving column (130 Å, 1.7 µm, 300 µm x 100 mm) using a 60 min gradient of 8 to 95 % acetonitrile in 0.1 % formic acid at 3 µL/min flow rate.

### DDA mass spectrometry

The eluted peptides were electro-sprayed into a Thermo Orbitrap Q Exactive HF Hybrid Quadrupole-Orbitrap Mass Spectrometer (ThermoFisher, USA). Data-dependent acquisition employed a top-40 ddMS2 method, where MS1 survey scans were over a range of 400-1200 m/z at a resolution of 60,000, and an AGC target of 3e6. The 40 most intense precursor ions were selected for fragmentation by high-energy collision-induced dissociation, and the resulting fragments were analyzed by MS2 over a range of 200-2000 m/z with a resolution of 15,000, isolation window of 2.0 m/z, AGC target of 1e6, and max IT of 40 ms. Dynamic exclusion was used within 30 s to prevent repetitive selection of the same peptide.

### DIA mass spectrometry

The eluted peptides were electro-sprayed into a Thermo nano-Eclipse Mass Spectrometer (Thermo Fisher, USA). The MS parameters were for full MS resolution of 120,000 with scan range at 390-1110 m/z. For MS2, the isolation window was 10.0 m/z, 70 scan events, resolution at 30,000.

### DDA mass spectrometry data analysis

DDA data analysis was performed using Thermo Fisher Proteome Discover software (version 2.5.0.400). Briefly, spectra were searched against the same previously generated *C. autoethanogenum* proteome database [1], with carbamidomethylation set as a fixed modification and up to 3 methionine oxidations. N-terminal acetylation, methionine loss and methionine loss and acetylation were also permitted. The mass tolerance of precursors and fragments were 10 ppm and 0.02 Da, respectively. The minimum and maximum peptide lengths were six and 144 amino acids, respectively. Two missed cleavages were allowed per peptide. Proteins were filtered to a 1% false discovery rate (FDR) cut-off threshold. For quantification, only peptides unique to a protein group were used. All other parameters were kept at their default settings. Proteins were identified as differentially expressed (DE) using cut-off thresholds of: abundance ratio adjusted p-value ≤ 0.05, q-value ≤ 0.05, number of unique peptides ≥ 2, and fold change (FC) ≥ 1.5 or ≤ 0.667 (i.e., |log₂FC | ≥ 0.585). FC quantification also required ≥ 2 samples per condition to have FDR ≤ 1% and therefore some comparisons showed “on/off” expression (when this was met for one condition but not the other).

### DIA mass spectrometry data analysis

Data analysis was performed with Spectronaut [2] version 15 (15.2.210819.50606) using direct DIA analysis and default settings. Briefly, spectra were searched against the *C. autoethanogenum* proteome database [1] with carbamidomethylation set as a fixed modification, methionine oxidation, and N-terminal acetylation as variable with 1% FDR cut-offs at the peptide spectral match, peptide and protein group levels. Quantitation was performed at the MS2 level with Q-value data filtering and cross run normalization with automatic row selection. GO term analysis was performed using a custom database generated from InterProScan [3,4]. Proteins were identified as differentially expressed (DE) using cut-off thresholds of: p-value ≤ 0.05, q-value ≤ 0.05, number of unique peptides ≥ 2, and fold change (FC) ≥ 1.5 or ≤ 0.667 (i.e., |log₂FC | ≥ 0.585).

# Appendix S2: Supporting gene variant analysis through alignment functions.

Most of *Clostridium autoethanogenum*’s gene annotations (and therefore genotype-phenotype relationships) are assumed from *Escherichia coli*, yeast, or other model organisms. BLAST® (BLASTX 2.13.0+) [5–7] and HHpred [8] searches were performed to obtain the best possible idea of what cellular function the variant genes could be involved in. HHpred can lead to greater prediction of real protein function, as its databases use more curated sequences (i.e., Protein Data Bank [PDB] have X-ray crystallography, NMR spectroscopy, or cryo-electron microscopy derived-structures), and the algorithm infers structural relationships from the amino acid sequence. However, some genes do not have strong matches to well-curated proteins, potentially making an HHpred search limited compared to BLASTX in this case. A summary of the search alignments is in Appendix S3 and based on these the following information was found.

**CAETHG_RS07040:** most similar to various types of ferric uptake transcriptional regulators (Fur), where zinc uptake transcriptional regulators (Zur) and peroxide stress response transcriptional regulators (PerR) are homologs within the same family (Appendix S3) [9,10]. **Evolved A, C & E - 86/140 histidine (H) → asparagine (N):** residue involved in binding regulatory metal ions and H is conserved for various organisms of differing phyla [9]. For their zinc uptake regulator, Liu et al. [11] found that this residue is one of four coordinating the regulatory zinc ion (i.e., zinc perception controls apo- and holo-form). However, alanine mutation of this ligand had the least effect on DNA binding (i.e., compared to the other three sites, “moderate” affect to regulation), believed to be due to other nearby (potential) zinc ligands. **Evolved C & E - 69/140 glutamic acid (E) → lysine (K):** residue contributes to DNA recognition, but is not highly conserved – K being more representative of Zur and E being more representative of PerR [11]. Alanine mutation of this residue significantly impaired DNA binding, but had a smaller impact than four other residues, which were more conserved (10 total residues) [11]. Although Fur/Zur/PerR can have various mechanisms of regulation, it seems likely that these mutations were in response to stress caused by the greater metabolic burden for reducing CO₂ compared to that of CO.

**CAETHG_RS07225:** spore germination protein, involved in transduction of sporulation through binding of small metabolites. Only the N-terminal domain structure has been elucidated, where the transmembrane and hydrophilic C-terminal domain structures are unresolved. **Evolved D - 513/525 Gln (Q) → Lys (K):** missense mutation is in the C-terminal hydrophilic region, where the functional difference between Q and K should be minimal (polar vs. positively charged sidechains).

**CAETHG_RS11655:** release factor 2 (RF2) promotes termination of protein synthesis through recognition of UAA and UGA stop codons. UGA occurs for ~15% of stop codons in C. autoethanogenum, UAA (~57%) and UAG (~28%) are more common and are recognized by RF1 (i.e., UGA is only recognized by RF2, UAA is shared, and UAG by RF1). **Evolved D - 325/368 Arg (R) → Ile (I):** R is a highly conserved residue on the C-terminus end of RF2’s switch loop [12], which undergoes a large conformational change during stop codon binding [13]. This flexibility is essential for positioning the GCQ motif for hydrolysis of the peptidyl-tRNA ester linkage in the peptidyl transferase center of the ribosome [13]. **Evolved E & G - 155/368 Arg (R) → *:** the mutation leads to a UGA stop codon, but for the protein to be truncated at least a transient amount of the native protein is required, where stop-codon readthrough or ribosome recuse are potential cellular mechanisms for translation of native protein [14,15]. Interestingly, this would lead to a translation negative-feedback response: over time there will be a shift from translation of native protein to truncated protein; when the functional protein diminishes (e.g., truncated protein predominates and/or native protein degrades), the native protein can be translated again. Therefore, it appears that the mutation would essentially lead to a reduction in functional RF2. Potentially, this would cause an increase in concentration for proteins without UGA stop codon, due to inferior translation of those terminated by UGA. Further, UGA readthrough has been linked to a variety of relevant aspects in E. coli, including faster growth from the stationary phase, and enhanced environmental adaption through increased phenotypic diversity in a genetically-identical population [16].

**CAETHG_RS13810:** homoserine kinase is involved in glycine, serine and threonine metabolism and catalyzes the transfer of the alcohol group from homoserine for a phosphate group. **Evolved D - 264/299 Asn (N) → Ser (S):** the mutation is in a non-conserved residue and does not appear to be key to thrB function. 264/299 appears to be part of an exterior loop away from the catalytic site. Further, S sidechain does not differ significantly from N. If the mutation did affect protein function though, it may shift the flux distribution around the reductive glycine pathway (RGP). The RGP has not been shown to be active in *C. autoethanogenum*, but the genes are present, and activity has been shown in closely related *Clostridium drakei* [16] and *Clostridium ljungdahlii* [17]. WLP flux during CO/CO₂/H₂ fermentation with *C. autoethanogenum* is double that of CO-dominant fermentations [18], potentially indicating its activity. Glycine is also consumed and slightly increases acetate production during heterotrophic growth (i.e., fructose vs. fructose + glycine), while threonine has a larger impact on acetate production [19], potentially due to acetaldehyde production during conversion to glycine. Two glycine cleavage system H proteins (CAETHG_RS07795 & 18030) and dihydrolipoyl dehydrogenase (CAETHG_RS07825) have increased expression in Evolved D, but two other glycine cleavage system proteins (CAETHG_RS02269 - gcvPA and CAETHG_RS02270 - gcvT) were not detected (only detected in transcriptomics of heterotrophic growth previously [19]). Enzymes involved in glycine conversion to pyruvate were detected with no change in expression (CAETHG_RS15910 - glyA and CAETHG_RS05870-5 - sda) and some enzymes involved in glycine conversion to acetyl-phosphate were detected (CAETHG_RS09165 - trxB) with increased expression (CAETHG_RS09170 - trxA). Other enzymes for glycine conversion to acetyl-phosphate are not annotated though (e.g., grd operon [17]). Further work is required to understand if a version of the reductive glycine pathway is active and what effect(s) mutations to thrB could have for the metabolism of C. autoethanogenum.

**CAETHG_RS14845:** this protein is highly similar to arginine repressors from various organisms. **Evolved A & G - 91/151 AsnPhe (NF) → TyrSer (TS):** these residues are somewhat conserved (as NL), where the L (or 92/151) is thought to be involved in hydrophobic interactions forming the trimeric core of the functional protein structure [20], which F is probably capable of fulfilling. Mutation of F to S will change a hydrophobic residue into a polar one though, more likely to create a non-functional or less-functional arginine repressor.

However, deleterious mutation of a large section of genome (at ~3.2785 Mb) was identified using the per-base coverage output from QualiMap BamQC analysis of sequence alignments (**Figure** **S1**). This type of mutation is likely to have resulted in a non-functional protein (**Table** **S3**) and as a significant deletion was identified for all Evolved lineages it is likely that mutation of this gene had a beneficial effect on CO₂/H₂ growth.

**References**

[1] K. Valgepea, R. de Souza Pinto Lemgruber, T. Abdalla, S. Binos, N. Takemori, A. Takemori, Y. Tanaka, R. Tappel, M. Köpke, S.D. Simpson, L.K. Nielsen, E. Marcellin, H2 drives metabolic rearrangements in gas-fermenting *Clostridium autoethanogenum*, Biotechnol. Biofuels. 11 (2018) 55. https://doi.org/10.1186/s13068-018-1052-9.

[2] R. Bruderer, O.M. Bernhardt, T. Gandhi, S.M. Miladinović, L.Y. Cheng, S. Messner, T. Ehrenberger, V. Zanotelli, Y. Butscheid, C. Escher, O. Vitek, O. Rinner, L. Reiter, Extending the limits of quantitative proteome profiling with data-independent acquisition and application to acetaminophen-treated three-dimensional liver microtissues, Mol. Cell. Proteomics. 14 (2015) 1400–1410. https://doi.org/10.1074/mcp.M114.044305.

[3] M. Blum, H.Y. Chang, S. Chuguransky, T. Grego, S. Kandasaamy, A. Mitchell, G. Nuka, T. Paysan-Lafosse, M. Qureshi, S. Raj, L. Richardson, G.A. Salazar, L. Williams, P. Bork, A. Bridge, J. Gough, D.H. Haft, I. Letunic, A. Marchler-Bauer, H. Mi, D.A. Natale, M. Necci, C.A. Orengo, A.P. Pandurangan, C. Rivoire, C.J.A. Sigrist, I. Sillitoe, N. Thanki, P.D. Thomas, S.C.E. Tosatto, C.H. Wu, A. Bateman, R.D. Finn, The InterPro protein families and domains database: 20 years on, Nucleic Acids Res. 49 (2021) D344–D354. https://doi.org/10.1093/nar/gkaa977.

[4] P. Jones, D. Binns, H.Y. Chang, M. Fraser, W. Li, C. McAnulla, H. McWilliam, J. Maslen, A. Mitchell, G. Nuka, S. Pesseat, A.F. Quinn, A. Sangrador-Vegas, M. Scheremetjew, S.Y. Yong, R. Lopez, S. Hunter, InterProScan 5: Genome-scale protein function classification, Bioinformatics. 30 (2014) 1236–1240. https://doi.org/10.1093/bioinformatics/btu031.

[5] S.F. Altschul, T.L. Madden, A.A. Schäffer, J. Zhang, Z. Zhang, W. Miller, D.J. Lipman, Gapped BLAST and PSI-BLAST: A new generation of protein database search programs, Nucleic Acids Res. 25 (1997) 3389–3402. https://doi.org/10.1093/nar/25.17.3389.

[6] Z. Zhang, S. Schwartz, L. Wagner, W. Miller, A Greedy Algorithm for Aligning DNA Sequences, J. Comput. Biol. 7 (2000) 203–214. https://doi.org/10.1089/10665270050081478.

[7] C. Camacho, G. Coulouris, V. Avagyan, N. Ma, J. Papadopoulos, K. Bealer, T.L. Madden, BLAST+: architecture and applications, BMC Bioinformatics. 10 (2009) 421. https://doi.org/10.1186/1471-2105-10-421.

[8] F. Gabler, S.Z. Nam, S. Till, M. Mirdita, M. Steinegger, J. Söding, A.N. Lupas, V. Alva, Protein Sequence Analysis Using the MPI Bioinformatics Toolkit, Curr. Protoc. Bioinforma. 72 (2020) 1–30. https://doi.org/10.1002/cpbi.108.

[9] B. Troxell, H.M. Hassan, Transcriptional regulation by Ferric Uptake Regulator (Fur) in pathogenic bacteria, Front. Cell. Infect. Microbiol. 4 (2013) 1–13. https://doi.org/10.3389/fcimb.2013.00059.

[10] F. Liu, Z. Su, P. Chen, X. Tian, L. Wu, D.J. Tang, P. Li, H. Deng, P. Ding, Q. Fu, J.L. Tang, Z. Ming, Structural basis for zinc-induced activation of a zinc uptake transcriptional regulator, Nucleic Acids Res. 49 (2021) 6511–6528. https://doi.org/10.1093/nar/gkab432.

[11] C. Ma, D. Kurita, N. Li, Y. Chen, H. Himeno, N. Gao, Mechanistic insights into the alternative translation termination by ArfA and RF2, Nature. 541 (2017) 550–553. https://doi.org/10.1038/nature20822.

[12] A. Korostelev, H. Asahara, L. Lancaster, M. Laurberg, A. Hirschi, J. Zhu, S. Trakhanov, W.G. Scott, H.F. Noller, Crystal structure of a translation termination complex formed with release factor RF2, Proc. Natl. Acad. Sci. U. S. A. 105 (2008) 19684–19689. https://doi.org/10.1073/pnas.0810953105.

[13] H. Zhang, Z. Lyu, Y. Fan, C.R. Evans, K.W. Barber, K. Banerjee, O.A. Igoshin, J. Rinehart, J. Ling, Metabolic stress promotes stop-codon readthrough and phenotypic heterogeneity, Proc. Natl. Acad. Sci. U. S. A. 117 (2020) 22167–22172. https://doi.org/10.1073/pnas.2013543117.

[14] C. Müller, C. Crowe-McAuliffe, D.N. Wilson, Ribosome Rescue Pathways in Bacteria, Front. Microbiol. 12 (2021) 1–20. https://doi.org/10.3389/fmicb.2021.652980.

[15] Y. Fan, C.R. Evans, K.W. Barber, K. Banerjee, K.J. Weiss, W. Margolin, O.A. Igoshin, J. Rinehart, J. Ling, Heterogeneity of Stop Codon Readthrough in Single Bacterial Cells and Implications for Population Fitness, Mol. Cell. 67 (2017) 826-836.e5. https://doi.org/10.1016/j.molcel.2017.07.010.

[16] Y. Song, J.S. Lee, J. Shin, G.M. Lee, S. Jin, S. Kang, J.K. Lee, D.R. Kim, E.Y. Lee, S.C. Kim, S. Cho, D. Kim, B.K. Cho, Functional cooperation of the glycine synthasereductase and Wood-Ljungdahl pathways for autotrophic growth of *Clostridium drakei*, Proc. Natl. Acad. Sci. U. S. A. 117 (2020) 7516–7523. https://doi.org/10.1073/pnas.1912289117.

[17] M.L. Dahle, E.T. Papoutsakis, M.R. Antoniewicz, 13C-metabolic flux analysis of *Clostridium ljungdahlii* illuminates its core metabolism under mixotrophic culture conditions, Metab. Eng. 72 (2022) 161–170. https://doi.org/10.1016/j.ymben.2022.03.011.

[18] J.K. Heffernan, K. Valgepea, R. de Souza Pinto Lemgruber, I. Casini, M. Plan, R. Tappel, S.D. Simpson, M. Köpke, L.K. Nielsen, E. Marcellin, Enhancing CO₂ -valorization using *Clostridium autoethanogenum* for sustainable fuel and chemicals production, Front. Bioeng. Biotechnol. 8 (2020) 204. https://doi.org/10.3389/fbioe.2020.00204.

[19] K. Valgepea, K.Q. Loi, J.B. Behrendorff, R. de S.P. Lemgruber, M. Plan, M.P. Hodson, M. Köpke, L.K. Nielsen, E. Marcellin, Arginine deiminase pathway provides ATP and boosts growth of the gas- fermenting acetogen *Clostridium autoethanogenum*, Metab. Eng. 41 (2017) 202–211. https://doi.org/10.1016/j.ymben.2017.04.007.

[20] Y.W. Park, J. Kang, H.K. Yeo, J.Y. Lee, Structural analysis and insights into the oligomeric state of an arginine-dependent transcriptional regulator from *Bacillus halodurans*, PLoS One. 11 (2016) 1–11. https://doi.org/10.1371/journal.pone.0155396.
